# Granulation compared to co-application of biochar plus mineral fertilizer and its impacts on crop growth and nutrient leaching

**DOI:** 10.1038/s41598-024-66992-0

**Published:** 2024-07-17

**Authors:** Jannis Grafmüller, Jens Möllmer, E. Marie Muehe, Claudia I. Kammann, Daniel Kray, Hans-Peter Schmidt, Nikolas Hagemann

**Affiliations:** 1 Institute of Sustainable Energy Systems (INES), Offenburg University of Applied Sciences, Offenburg, Germany; 2 Ithaka Institute, Arbaz, Switzerland; 3 Ithaka Institute, Goldbach, Germany; 4 Plant Biogeochemistry, Department of Geosciences, University of Tübingen, Tübingen, Germany; 5 Environmental Analytics, Agroscope, Zurich, Switzerland; 6 Institut für Nichtklassische Chemie e.V. (INC), Leipzig, Germany; 7 Plant Biogeochemistry, Department of Applied Microbial Ecology, Helmholtz Centre for Environmental Research - UFZ, Leipzig, Germany; 8 Department of Applied Ecology, Hochschule Geisenheim University, Geisenheim, Germany

**Keywords:** Biochar-based fertilizer, BBF, Pyrolysis, PyCCs, White cabbage, Environmental impact, Crop waste

## Abstract

Mechanized biochar field application remains challenging due to biochar’s poor flowability and bulk density. Granulation of biochar with fertilizer provides a product ready for application with well-established machinery. However, it’s unknown whether granulated biochar-based fertilizers (gBBF) are as effective as co-application of non-granulated biochar with fertilizer. Here, we compared a gBBF with a mineral compound fertilizer (control), and with a non-granulated biochar that was co-applied at a rate of 1.1 t ha^−1^ with the fertilizer in a white cabbage greenhouse pot trial. Half the pots received heavy rain simulation treatments to investigate nutrient leaching. Crop yields were not significantly increased by biochar without leaching compared to the control. With leaching, cabbage yield increased with gBBF and biochar-co-application by 14% (*p* > 0.05) and 34% (*p* < 0.05), respectively. Nitrogen leaching was reduced by 26–35% with both biochar amendments. Biochar significantly reduced potassium, magnesium, and sulfur leaching. Most nitrogen associated with gBBF was released during the trial and the granulated biochar regained its microporosity. Enriching fertilizers with biochar by granulation or co-application can improve crop yields and decrease nutrient leaching. While the gBBF yielded less biomass compared to biochar co-application, improved mechanized field application after granulation could facilitate the implementation of biochar application in agriculture.

## Introduction

Biochar is being considered for application in agriculture to improve crop yields and make soils more resilient to global warming induced challenges while creating long-term carbon sinks. Biochar can be implemented in different agricultural practices, e.g., as a soil amendment^1,2^, feed supplement^3^, compost additive^4^, or as a component of fertilizers to reduce N losses from soils^5^. The combination of biochar and fertilizer is known as biochar-based fertilizer (BBF)^6^. A recent meta-analysis found that BBFs increased crop productivity by 10% compared to fertilized controls without biochar amendment^6^. This might be due to improved retention of fertilizer by biochar in soil, which can lead to increased nutrient use efficiencies and higher crop yields compared to conventional fertilizers without biochar^2,7–10^.

A BBF is produced through (1) sorption of nutrients by biochar from a liquid^11–14^, (2) infusing nutrients into biochar by heating a mixture of biochar and fertilizer under controlled conditions^15^, (3) coating of solid, granulated fertilizers with biochar^7^, or (4) pelleting and granulation of biochar with solid nutrient-rich materials^8,16^. Granulation of biochar and fertilizer results in a granulated BBF (gBBF) with a homogeneous particle size distribution and improved flowability, which eases mechanized application to soil. With that, current challenges during application of biochar with common agricultural machinery might be overcome, such as blockages of fertilizer spreaders due to the inhomogeneity in biochar particle size and its low bulk density.

However, to the best of our knowledge, no study so far investigated whether gBBF is as effective as the co-application of non-granulated (and non-milled) biochar and fertilizer to soil. Granulation processes require raw material particle sizes below 300–500 µm, i.e., biochar must be milled. Additionally, granulation reduces the number of particles per mass unit of biochar resulting in fewer particles being applied to a volume of soil. Thus, there is less direct exchange interface between biochar and soil as long as the granules retain their shape. Both effects could affect plant growth in comparison to the application of non-granulated biochar. In addition, granulation with fertilizer can reduce biochar’s porosity^17^, which is an important parameter for the interaction of biochar with soil nutrients. However, it is unknown how porosity of gBBF evolves after soil application. Still, the leaching of nutrients from soils amended with a gBBF might be reduced compared to the co-application of biochar and fertilizer, as the interaction between nutrients and biochar could be enhanced to a higher extent in granulated gBBFs^10^.

In the present study, a gBBF was produced from milled biochar (< 1 mm) using 0.02% (w/w) of binding agent (carboxymethyl cellulose), which is considerably lower than in some earlier studies^7–9,15–17^ and allows to study the impact of the biochar’s granulation on crop growth and nutrient leaching without secondary effects of such additives. In addition, biochar was loosely mixed into the soil along with the granulated NPK fertilizer (B + NPK), reflecting the most frequent pathway of biochar uses in agriculture (co-application). For this purpose, biochar was milled to < 12 mm for practical reasons, but not finely milled as for granulation (collard mill, < 1 mm). Both treatments were compared to an NPK-fertilizer control without biochar (NPK, Fig. S1). The aim was to assess how the granulation process impacts the effects of biochar on crop growth and nutrient leaching, i.e., if B + NPK would differ from gBBF. We hypothesized that gBBF would improve nutrient retention and crop growth more effectively than co-applied B + NPK, due to the close contact of biochar and nutrients in gBBF. We assumed that granulation may allow enhanced nutrient sorption onto biochar surfaces and into biochar pores. Furthermore, we studied the changes in porosity that the biochar incorporated in the gBBF underwent after soil application.

## Materials and methods

### Production of fertilizers

Biochar certified according to the European Biochar Certificate (EBC)^18^ was obtained from an industrial pyrolysis plant using untreated wood chips (Carbon Cycle GmbH & Co. KG, Rieden, Germany, https://www.european-biochar.org/cert/4py5-h7f2-xwj6-v6w8/en) at a maximum pyrolysis temperature of 750 °C. Biochar was processed in a hammer mill to < 12 mm and further milled to < 1 mm in a collard mill (Type SJM00F, Gebr. G. Fischer AG, Schaffhausen, Switzerland). For the production of gBBF, biochar (13.3 kg, < 1 mm), 3.4 kg urea (N source, without urease and nitrification inhibitors), 1.2 kg mono-potassium phosphate (phosphorus (P) and potassium (K) source), and 2.5 kg Patentkali® (P, K, magnesium (Mg) and sulfur (S) source, all of technical purity grade, dry matter equivalents) were mixed in a granulator (Type SK G1, Gustav Eirich GmbH, Hardheim, Germany). Water (4 kg) followed by 0.6 kg of a 0.8% (w/w) solution of hydroxypropyl methyl cellulose (Arbocel^®^ CE2910 HE 50 LV, J. Rettenmaier & Söhne GmbH + Co. KG, Rosenberg, Germany) were stepwise added to the biochar-nutrient mix via a sprayer nozzle, which resulted in a binding agent concentration of 0.02% (w/w) in the end product. The granulated fertilizer without the addition of biochar was produced under otherwise unchanged conditions but with less water addition, keeping the binder-to-nutrient ratio constant, and was labeled as NPK. After the particles had agglomerated to the desired granule size range, the product was sieved to 2–4 mm and dried at 90 °C for 90 min to reach sufficient mechanical stability. Both types of granulated fertilizers and the pure biochar (< 12 mm milling fraction) were packed in air-tight bags and stored under ambient conditions in the dark until further usage.

### Basic characterization of granulated fertilizers and biochar

Biochar, gBBF, and NPK were analyzed for total carbon, nitrogen, hydrogen, and sulfur content (CHNS), macro and microelements, and ash content according to the EBC analytical guidelines^18^ by a commercial lab (Eurofins Umwelt Ost GmbH, Bobritzsch-Hilbersdorf, Germany). The pH of biochar, gBBF, and NPK was measured after shaking for 1 h in 0.01 M CaCl_2_ (1:10, w/w). The porosity of pristine biochar (< 1 mm after collard milling), gBBF, and NPK was characterized by recording CO_2_ and N_2_ adsorption isotherms on an Autosorb iQ (Quantachrome Instruments, Anton Paar GmbH, Ostfildern-Scharnhausen, Germany), as described in section 1.1 of the SI. Specific surface area (SSA) was calculated using either the Brunauer–Emmett–Teller^19^ (BET) method or the density functional theory^20^ (DFT). Scanning Electron Microscopy (SEM) and electron dispersive X-ray (EDX) mapping were performed for gBBF as described in section 1.2 of the SI.

### N release from fertilizers

The release of N from NPK, gBBF, and B + NPK was determined by repeated extraction with CaCl_2_ and incubation in distilled water. For the repeated extraction, samples were weighed into 100 mL Erlenmeyer flasks (equivalent of 0.4 g of N per flask), where 50 mL of a 0.0125 M CaCl_2_ solution was added. The flasks were shaken at 125 rpm on a horizontal shaker for 1 h. After decanting and addition of fresh extractant to the sample, a second and third extraction step was performed (with shaking for a further 2 h and 45 h, respectively; for details see section 1.3 in the SI). Filtered extracts (< 0.45 µm) were quantified for total dissolved nitrogen (TN) by chemiluminescence as described below in the “Analysis of N species in fertilizer extracts, leachates, and soil extracts” section. Extracted TN was calculated after each extraction step as described in section 1.3 of the SI.

For the incubation study, fertilizers (equivalent to 0.4 g N) were incubated in 100 mL of de-ionized water in triplicates in Schott bottles at 20 °C without shaking as was suggested by Liu et al.^15^. After 1, 3, 6, 20, 24, 120, and 220 h, 5 mL liquid was sampled and filtered to < 0.45 µm and de-ionized water (5 mL) was added to the bottle to maintain a constant solid-to-liquid ratio. Samples were measured for dissolved TN and release of N was calculated as detailed in the SI (section 1.3).

### Greenhouse pot experiment

#### Origin and analysis of the soil used for the pot experiment

Sandy topsoil (0–20 cm) with a pH of 7.4 and organic matter content of 7.7% was excavated in March 2022 from an arable field in the Ortenau Region near river Rhein (48° 32′ 23.7″ N, 7° 48′ 50.6″ E, Kehl-Sundheim, Germany), sieved to < 10 mm and thoroughly homogenized before taking a representative subsample for the analysis^21^ at Eurofins Umwelt-Ost GmbH (Jena, Germany, Tables S2–S4). The soil was stored under dry conditions for 3 months in air-permeable plastic bags until further usage. The amount of soil used for the pot experiment was re-homogenized before the experiment.

#### Pot setup and management

Pots with a volume of 4 L (17 cm inner diameter at the top, 21 cm height) equipped with a 2 mm Nylon mesh at the bottom were filled with a homogeneous mixture of 4000 g dry matter equivalent of the soil and 3.5 g N in one of the three different fertilizer types: (1) NPK, (2) gBBF and (3) B + NPK (Table 1). This corresponds to 140 kg N ha^−1^, assuming a plant density of 40,000 plants ha^−1^. Detailed information on resulting nutrient dosage (P, K, Mg, S) per pot can be found in Table S5. The biochar application rates in the gBBF and B + NPK treatments were both 26.7 g dry weight pot^−1^, corresponding to 1.1 t biochar ha^−1^ assuming the above-mentioned plant density. Five replicate pots per treatment were either prepared for cultivation with or without leaching events, the latter including a non-fertilized control (CTRL-0) to test for leaching of native soil nutrients and mineralized N (Table 1). A control group without fertilizer/biochar application and without leaching events was not conducted as a pre-trial showed that no cabbage head development could be expected^13^. Five additional pots for NPK, gBBF, and B + NPK without leaching were set up for repeated sampling to measure soil N speciation and urease activity over time (cf. “Soil sampling and analysis” section). In total 50 pots were set-up. In the middle of each soil-filled pot, two seeds of white cabbage were sown on 5th of August 2022 and reduced to one plant per pot two weeks after sowing (cabbage variety Sunta F1, Bruno Nebelung GmbH, Everswinkel, Germany). Pots were arranged in a randomized block design on a greenhouse table. Soils were kept at 65% water holding capacity (WHC), further details on trial maintenance can be found in section 1.4 of the SI.

**Table 1:** Treatments prepared for the greenhouse trial.

Treatment ID	Treatment	Leaching
1	NPK	None
2	gBBF	None
3	B + NPK	None
4	CTRL-0	30 L m^−2^ (twice)
5	NPK	30 L m^−2^ (twice)
6	gBBF	30 L m^−2^ (twice)
7	B + NPK	30 L m^−2^ (twice)

NPK: granulated, mineral nitrogen, phosphorus and potassium fertilizer. gBBF: granulated biochar-based NPK fertilizer. B + NPK: co-application biochar and granulated NPK fertilizer to the soil. CTRL-0: non-fertilized control.

#### Leaching events

The simulation of extreme rain events was performed 35 and 63 days after sowing in treatments ID4–ID7 (cf. Table 1). Pots were irrigated with tap water to achieve 65% WHC and placed on a pipe socket with a sealed bottom (Fig. S5). For each pot, a total of 0.7 L of distilled water was added within 60 min in steps of 0.1 L every 8 min, which corresponds to an extreme precipitation event^22^ of 30 mm calculated based on the soil surface of the pot. 30 min after the simulated rain event, leachate samples were filtered to < 0.45 µm and immediately stored on ice and analyzed for nitrate–N (NO_3_^-^-N), ammonium–N (NH_4_^+^-N) and total dissolved N (TN) as described in the “Analysis of N species in fertilizer extracts, leachates, and soil extracts” section. After storage at − 20 °C, the samples were also analyzed for P, K, Mg and S by Inductively Coupled Plasma Optical Emission Spectroscopy (ICP-OES, icap 7000 series, Thermo Scientific, Waltham, USA).

#### Biomass harvest and analysis

Cabbage plants were harvested 116 days after sowing by cutting the stem below the lowest leaf base. The fresh weight of total aboveground biomass and the cabbage head alone was recorded after removing outer protruding leaves (Fig. S6). All the harvested biomass was rasped, mixed and an aliquot of 50 g was dried to mass constancy at 80 °C for further analysis and to determine dry matter content. Roots were excavated, washed and dried at 80 °C to determine dry weights. The dried aboveground biomass was ground to < 1 mm (Retsch ZM 200) and measured for total N contents (CN928, LECO Corporation, St. Joseph, USA). The content of other main nutrients (P, K, Mg, S, Ca) and trace elements (Mn, Cu, Zn) was measured using ICP-OES after microwave digestion of the biomass (Mars 5 Xpress, CEM GmbH, Kamp-Lintfort, Germany, cf. section 1.5 of the SI for details). Nutrient uptake in the aboveground cabbage biomass was calculated by multiplying the measured nutrient contents in cabbage tissues with the aboveground biomass yield.

#### Soil sampling and analysis

Soil samples were taken with a soil core sampler (10 mm diameter) in five redundantly prepared pots for the NPK, gBBF, and B + NPK treatments without leaching 35 and 70 days after sowing for the whole soil depth (21 cm, Table 1, Fig. S4). Samples were stored at − 20 °C for analysis of extractable N species (NH_4_^+^-N, NO_3_^-^-N and TN) and at 4 °C for quantification of soil urease activity^23^. After harvest, the soil separated from the rootstock was mixed manually, representatively sampled (50 mL), and stored at − 20 °C for analysis of extractable N species and at 4 °C for quantification of soil urease activity. For soluble N extraction, 5 g of soil (fresh weight) was extracted in 20 mL 0.0125 M CaCl_2_ in closed 100 mL-Erlenmeyer flasks for 1 h on a rotary shaker at 125 rpm, based on DIN 19746^24^. The suspension was filtered to < 0.45 µm and stored at -20 °C if not measured directly for NH_4_^+^-N, NO_3_^-^-N, and TN content as described in the “Analysis of N species in fertilizer extracts, leachates, and soil extracts” section and reported based on soil dry matter. Soil pH values were measured according to DIN EN 15,933 on 5 mL air-dried samples in 25 mL 0.01 M CaCl_2_ (Titroline alpha Plus, SI Analytics GmbH, Mainz, Germany).

#### Extraction of residual nitrogen and SSA of soil-aged gBBF granules

Individual intact granules of gBBF (Fig. S7) found in the remaining soil of each pot after harvest and representative soil sampling were sampled and stored both for analysis at 4 °C (for porosity measurements) and − 20 °C (for residual N content). From each pot, a mass of 0.3–0.5 g (dry weight) of sampled granules was transferred to 100 mL Erlenmeyer flasks and dried to mass constancy at 55 °C. Samples were subsequently extracted with 20 mL of 2 M KCl for 2 h on a horizontal shaker with 150 rpm at room temperature. The suspension was filtered to < 0.45 µm and analyzed for TN as described in the “Analysis of N species in fertilizer extracts, leachates, and soil extracts” section. The SSA of soil-aged gBBF granules (dried at 40 °C, but otherwise untreated after extraction from soil) sampled from three individual pots per treatment were characterized by CO_2_ adsorption as described in the “Basic characterization of granulated fertilizers and biochar” section.

### Analysis of N species in fertilizer extracts, leachates, and soil extracts

NH_4_^+^-N concentrations in the leachates, soil, and fertilizer extracts were quantified with a Berthelot reaction according to Rhine and colleagues^25^ on 96 well microtiter plates with a microplate reader (Epoch2, Biotek Instruments, Winooski, USA). For NO_3_^-^-N quantification, a microplate reader method adapted from Hagemann et al.^26^ was applied using UV transparent 96 well microplates (UV-Star^®^, Greiner Bio-One GmbH, Frickenhausen, Germany). Total dissolved N in the leachates and extracts were quantified by chemiluminescence on a TOC-VCPN equipped with the TN measurement unit TNM-1 (Shimadzu Corporation, Kyoto, Japan). The difference between TN and the sum of NO_3_^-^-N and NH_4_^+^-N was defined as organic N (N_org_). Analytical techniques are described in more detail in section 1.6 of the SI.

### Data analysis

All statistical analyses were performed with Graphpad Prism (version 10.0.3, GraphPad Software LLC, Boston, USA). For biomass yields and nutrient uptakes, two-way analysis of variance (ANOVA) was performed using the factors fertilizer type (NPK/gBBF/B + NPK) and leaching (no leaching/including leaching) followed by Tukey’s post-hoc test at alpha = 0.05. For nutrient leaching, one-way ANOVA was performed, followed by Tukey’s post-hoc test to identify significant differences between different treatments at alpha = 0.05. Block effects were taken into account via the repeated measures function in Graphpad Prism.

## Results

### Fertilizer composition and morphology

The biochar used in the experiment had a molar H/C ratio of 0.2, a low ash content of 2% (w/w), and a low content of macronutrients like K or Mg (Table 2). Trace metal contents in the biochar were below limit values of the certification class AgroBio of the European Biochar Certificate^18^ (Table S7). The NPK fertilizer granule contained the desired amounts of N, P, and K (Table 2), and low concentrations of trace metals (Table S7). As the biochar was added at 65% (w/w) to the fertilizer to prepare the gBBF, the contents of the main elements and trace metals in gBBF were accordingly lower (Tables 2 and S7).

**Table 2:** Elemental analysis (carbon (C), hydrogen (H), nitrogen (N), and sulfur (S)), and contents of macronutrients (phosphorus as P_2_O_5_, potassium as K_2_O, and magnesium as MgO) in biochar and fertilizers.

Sample	C (%)	H (%)	N (%)	S (%)	Ash (%)	pH	P_2_O_5_ (gkg^−1^)	K_2_O (gkg^−1^)	MgO (gkg^−1^)	BET SSA^a^ (N_2_) (m^2^ g^−1^)	BET SSA^a^ (CO_2_) (m^2^ g^−1^)	DFT SSA^b^ (CO_2_) (m^2^ g^−1^)
Biochar	93.3	1.4	0.31	< 0.03	2.0	9.1	0.6	2.5	1.1	21^d^	358^d^ ± 43	516^d^ ± 52
NPK	10.2	3.7	23.0	6.3	46.3	5.2	85.2	159.0	31.5	7	42	n.a.^c^
gBBF	61.4	2.3	8.74	2.63	19.1	6.1	33.6	62.9	11.4	5	155 ± 26	116 ± 8

Specific surface area (SSA) of the biochar, the pure NPK fertilizer (NPK) and the granulated biochar-based NPK fertilizer (gBBF). Errors indicate the range of minimum to maximum measured values of n = 2 measurements, where applicable.

^a^BET SSA: Specific surface area calculated with the Brunauer–Emmet–Teller (BET) method based on either N_2_ or CO_2_ adsorption isotherms.

^b^DFT SSA: Specific surface area calculated with the density functional theory (DFT) based on CO_2_ adsorption isotherms.

^c^n.a.: not applicable since the adsorption isotherm did not show a Type-I isotherm characteristic.

^d^Biochar after milling with a collard mill.

The biochar had a microporous character (pore width ≤ 1.5 nm) and was low in mesopores (pore width 1.5 nm < × < 50 nm), which was indicated by (1) a steep increase in adsorbed volume of CO_2_ at low relative pressures in the CO_2_ adsorption isotherms and (2) only low adsorption amounts of N_2_ leading to a BET and DFT SSA of 358 m^2^ g^−1^ and 516 m^2^ g^−1^, respectively, when based on CO_2_ adsorption compared to only 20 m^2^ g^−1^ of BET SSA when based on N_2_ adsorption (Table 2, Figs. S8a and S9a). The CO_2_-based SSA of the gBBF was only 155 m^2^ g^−1^ (BET) and 116 m^2^ g^−1^ (DFT, Table 2). Three distinct pore sizes of approximately 0.35 nm, 0.5 nm and 0.8 nm were identified in the biochar, using the first derivate of the cumulative pore volume (calculated via DFT, Fig. S10). The pore size distribution of gBBF granules was of more multimodal character, with a decrease in the peak at 0.35 nm, a clear widening of the distribution at around 0.5 nm, and a higher contribution by pore widths at around 0.8 nm compared to the pure biochar (Fig. S10). The wider and multimodal pore size distribution at around 0.5 nm in gBBF reflected the more heterogeneous matrix compared to pure biochar, as the NPK fertilizer had a similar pore size distribution in that region (Fig. S10). The relative increase in pores with 0.8 nm width in gBBF was clearly linked with the added mineral fertilizer, which harbors a relatively high presence of pores in that dimension (Fig. S10).

Elemental mapping with EDX suggested that the nutrients in gBBF were homogeneously distributed onto the carbonaceous surfaces of the biochar, which was demonstrated by imaging the cross-section of individual granules after slicing with a scalpel (Figs. S12–S15). Furthermore, the EDX mappings indicated that some pores of the biochar in gBBF were filled with mineral nutrients from the added fertilizer (Fig. S14) and that individual biochar particles were compressed and embedded in each other (Fig. S11).

### Nitrogen release from granulated fertilizers and fertilizer-biochar mixture

**Figure 1:**
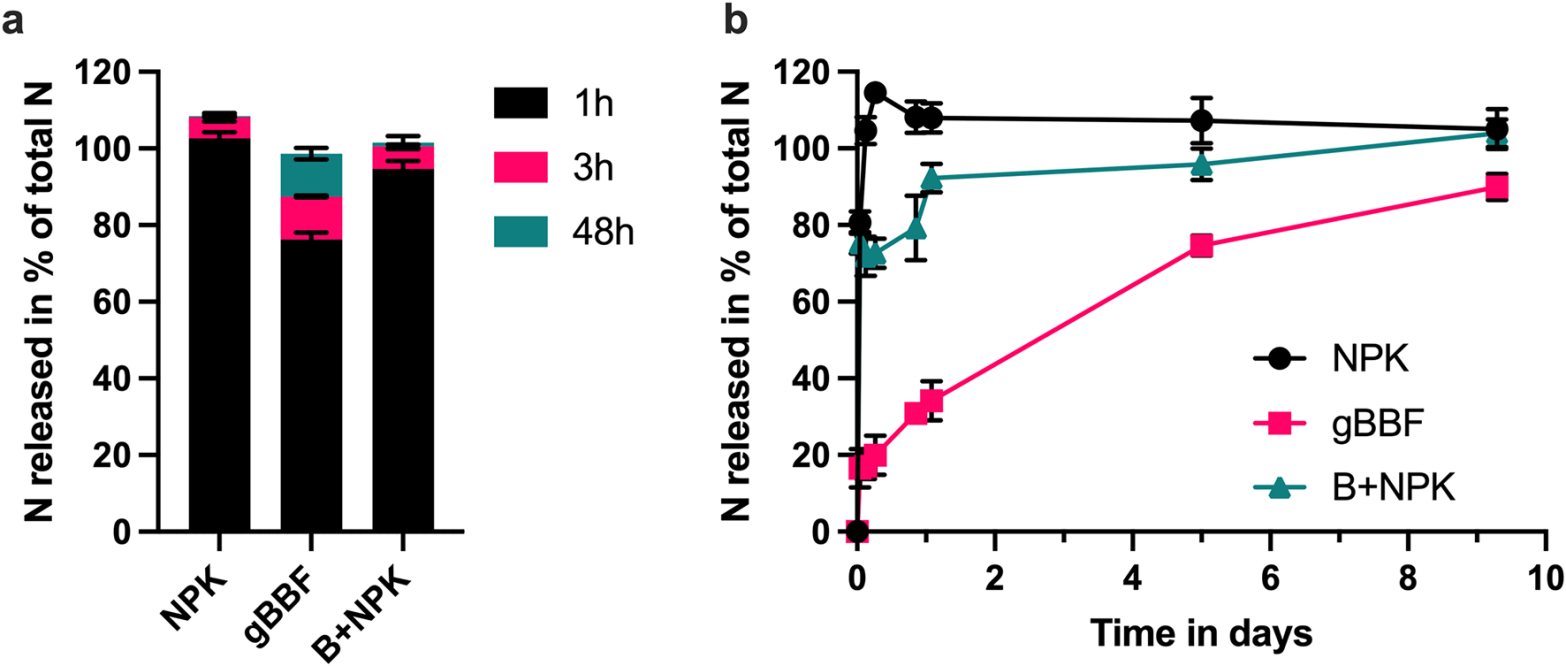
Nitrogen released from granulated NPK fertilizer (NPK) and granulated biochar-based fertilizer (gBBF) and the mixture of NPK with non-granulated biochar (B + NPK) in repeated extractions with 0.0125 M CaCl_2_ during the indicated time intervals (**a**), and during incubation in distilled water for nine days (**b**). Data are presented as means ± standard deviation (n = 3). Released N is presented as a percentage of the total N contained in the different fertilizers.

In repeated extractions with 0.01 M CaCl_2_, NPK and B + NPK released virtually all N during the first extraction step of 1 h with 102% and 95%, respectively (Fig. 1a). For the gBBF, the first extraction step only liberated 76% of total N, followed by extraction of 11% each after 3 and 48 h (Fig. 1a). A slower release of N from the gBBF compared to NPK and B + NPK was also observed in the liquid incubation experiment without continuous shaking of the incubation medium (Fig. 1b). While NPK and B + NPK released 100% and 92% of N to the liquid during the first day of incubation, respectively, gBBF only released 34% within the same period of time (Fig. 1b). The release of N from gBBF to the incubation liquid was significantly lower during the whole incubation period compared to the other treatments. For gBBF, 90% of N-release was achieved after nine days of incubation (*p* < 0.05). It has to be noted that biochar in B + NPK was milled to < 12 mm, while gBBF was produced after additional milling of biochar to < 1 mm. However, nutrient release from mixtures of biochar and NPK was not affected by particle sizes within the range relevant to this study, as detailed in the SI (Fig. S16).

### Biomass yield and nutrient uptake

**Figure 2:**
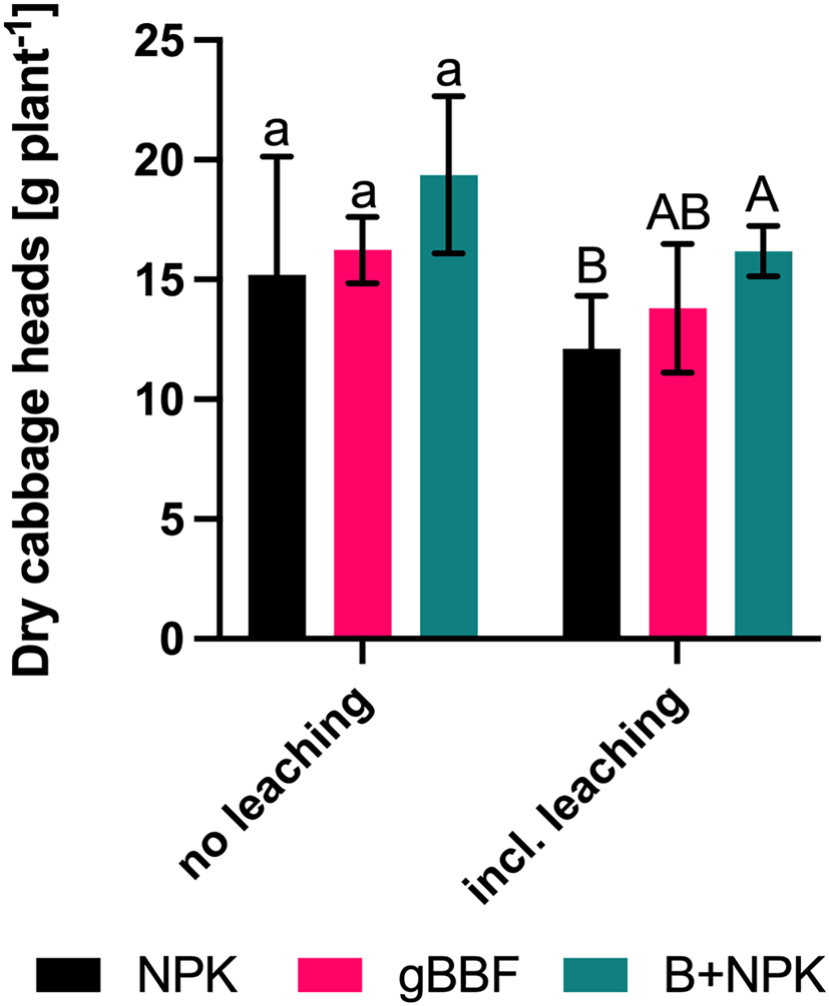
Yields of dry cabbage heads for the different fertilizer types for plants that were grown without leaching events (no leaching) or with two leaching events (incl. leaching, each 30 L m^−2^). NPK: granulated, mineral nitrogen, phosphorus and potassium fertilizer. gBBF: granulated biochar-based NPK fertilizer. B + NPK: co-application of non-granulated biochar and granulated NPK fertilizer to the soil. Data are presented as means ± standard deviation (n = 5). Different letters above each error bar indicate a significant difference between treatments without or with leaching events (lowercase and uppercase letters, respectively, two-way analysis of variance and Tukey’s post-hoc test at *p* < 0.05).

Dry cabbage head yields ranged between 15 and 20 g plant^−1^ in treatments without leaching events and between 12 and 16 g plant^−1^ for the same treatments including leaching events (Fig. 2). Leaching decreased cabbage head yields compared to the equally fertilized plants grown in the absence of leaching (Fig. 2, Table 4, *p* = 0.06). Fertilizer type did not significantly affect dry cabbage head yields when plants were cultivated without leaching events (Fig. 2). Still, B + NPK tended to increase cabbage head yield compared to NPK (+ 27%, *p* = 0.07). For the plants grown with leaching events, the amendment of B + NPK significantly increased dry cabbage head yields by 34% relative to the NPK treatment (*p* < 0.05), while the increase under gBBF relative to NPK was not statistically significant (*p* = 0.53, Fig. 2).

Total aboveground biomass yields mirrored the yields of cabbage heads. The amendment of B + NPK tended to increase dry aboveground biomass without leaching events and significantly increased it by 19% compared to NPK in the presence of leaching (*p* < 0.05, Tables 3 and 4). Dry root biomass was significantly affected by the fertilizer type in absence of leaching events (Tables 3 and 4). The NPK treatment yielded the highest root biomass of 1.6 g plant^−1^, while the B + NPK treatment had the lowest with 1.1 g plant^−1^ (*p* < 0.0 Table 3). Root growth with gBBF was with 1.3 g plant^−1^ in between these two different treatments (Table 3). Leaching events reduced root biomass to 0.9–1.0 g plant^−1^ for the NPK and gBBF treatments, but remained unchanged with B + NPK (Tables 3 and 4).

**Table 3:** Aboveground biomass and root biomass of cabbage plants.

Treatment	Fresh total aboveground biomass in g	Fresh cabbage heads in g	Dry total aboveground biomass in g	Dry root biomass in g
**No leaching events**				
NPK	376 ± 47 a	232 ± 61 a	24.8 ± 3.1 a	1.6 ± 0.2 a
gBBF	380 ± 10 a	250 ± 13 a	24.7 ± 1.0 a	1.3 ± 0.3 ab
B + NPK	418 ± 21 a	281 ± 14 a	28.8 ± 3.8 a	1.1 ± 0.1 b
**Incl. leaching events**				
NPK	340 ± 12 B	188 ± 33 B	21.9 ± 1.6 B	1.0 ± 0.1 A
gBBF	363 ± 34 AB	211 ± 42 AB	23.8 ± 1.7 AB	0.9 ± 0.1 A
B + NPK	395 ± 9 A	246 ± 17 A	26.1 ± 1.8 A	1.1 ± 0.1 A

NPK: granulated mineral nitrogen (N), phosphorus (P) and potassium (K) fertilizer. gBBF: granulated biochar-based NPK fertilizer. B + NPK: co-application of non-granulated biochar and NPK fertilizer to the soil. Data are presented as means ± standard deviation (n = 5). Different lowercase letters within a row indicate significant differences between the treatments cultivated without leaching events. Different uppercase letters indicate significant differences between the treatments including two leaching events (two-way analysis of variance, *p* < 0.05, followed by Tukey’s post-hoc test).

**Table 4:** Statistical results of two-way analyzes of variance of dry matter aboveground and belowground biomass yields.

Final biomass harvest	Total biomass		Cabbage heads		Roots	
Factor	F	*p*	F	*p*	F	*p*
Leaching	3.61	0.099	4.89	0.063	10.91	**0.013**
Fertilizer type	5.58	**0.017**	6.56	**0.010**	3.181	0.073
Leaching × fertilizer type	0.11	0.898	0.06	0.941	9.017	**0.003**
Block	1.39	0.282	1.90	0.141	1.869	0.151

Factors: ‘Leaching’ (no leaching /incl. leaching), ‘Fertilizer type’ (NPK/gBBF/B + NPK) and the interaction of both individual factors. Additionally, the block effect is presented ‘Block’.

Significant values are displayed in bold.

**Figure 3:**
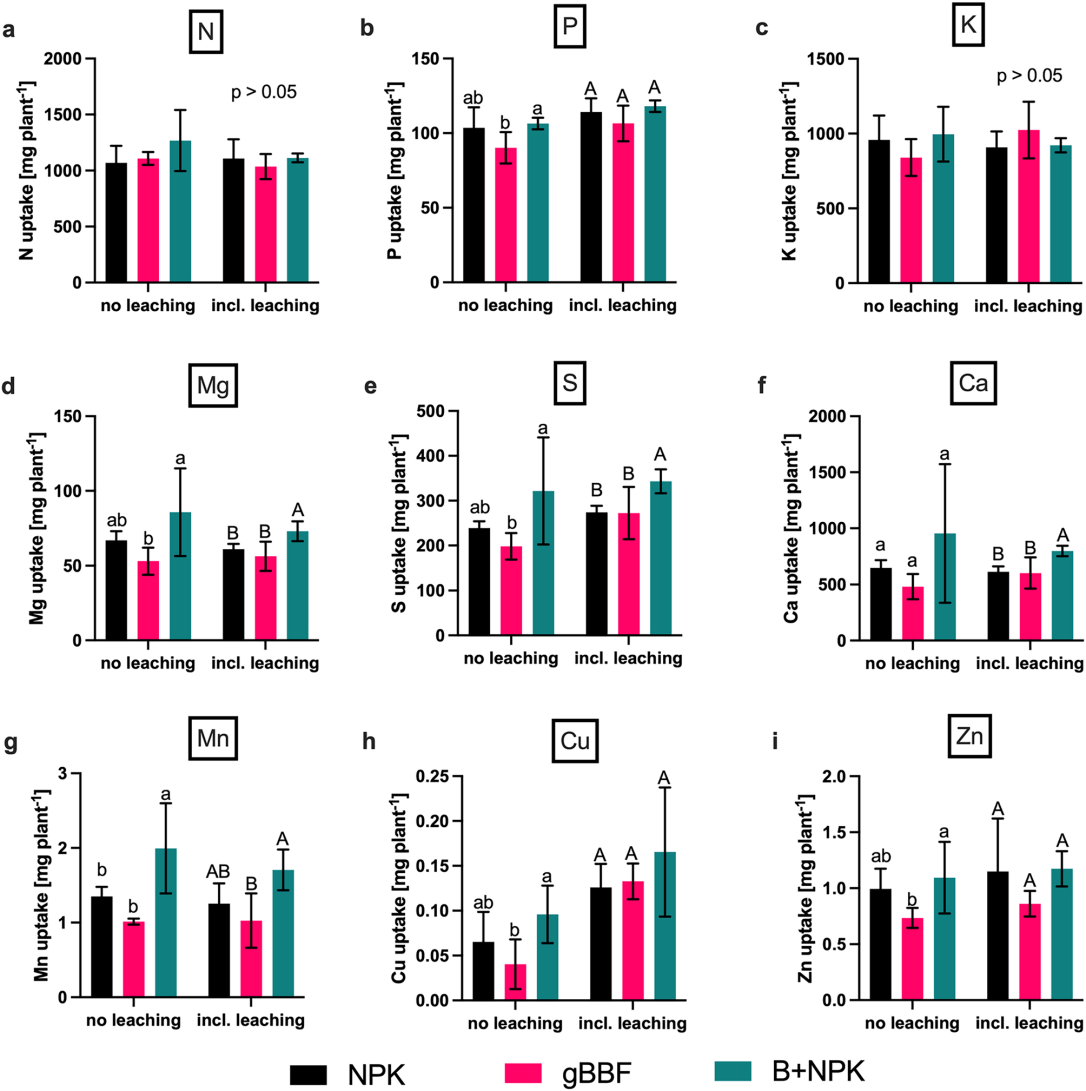
Nutrient uptake in aboveground cabbage biomass cultivated without (no leaching) or with two leaching events (incl. leaching, 30 L m^−2^ each 35 and 63 days after sowing): nitrogen (N), potassium (K), phosphorus (P), magnesium (Mg), sulfur (S), calcium (Ca), manganese (Mn), copper (Cu) and zinc (Zn). NPK: granulated, mineral fertilizer. gBBF: granulated biochar-based NPK fertilizer. B + NPK: co-application of non-granulated biochar and granulated NPK fertilizer to the soil. Data are presented as means ± standard deviation (n = 5). Different letters above error bars indicate a statistically significant difference within the no leaching or the incl. leaching treatments (lowercase and uppercase letters, respectively, two-way analysis of variance and Tukey’s post-hoc test, *p* < 0.05).

Nitrogen uptake in the aboveground biomass was in the range of 1.0–1.2 g plant^−1^ and it was not significantly affected by the leaching treatment nor the fertilizer type (Fig. 3a, Table S10). Nitrogen use efficiencies ranged between 30 and 35% for all plants, based on N uptakes into the aboveground biomass related to the applied N fertilizer (Fig. S20). Without leaching events, B + NPK significantly increased P, Mg, S, Mn and Cu uptakes compared to the gBBF treatment, and for Mn also compared to the NPK treatment (*p* < 0.05, Fig. 3). The lower uptakes of these nutrients with gBBF under no-leaching conditions were not only a result of lower aboveground biomass yields with gBBF compared to B + NPK (Table 3), but also due to lower contents for most of these nutrients in the aboveground cabbage tissues (Fig. S17). With the leaching treatment, B + NPK significantly increased the uptakes of Mg, S, Ca, and Mn compared to NPK and gBBF (*p* < 0.05, Fig. 3). Moreover, leaching significantly increased the plants’ uptake of P, S and Cu compared to the plants grown without leaching, independent of the fertilization treatment (Table S10, Fig. 3).

### Nutrient leaching from planted pots

**Figure 4:**
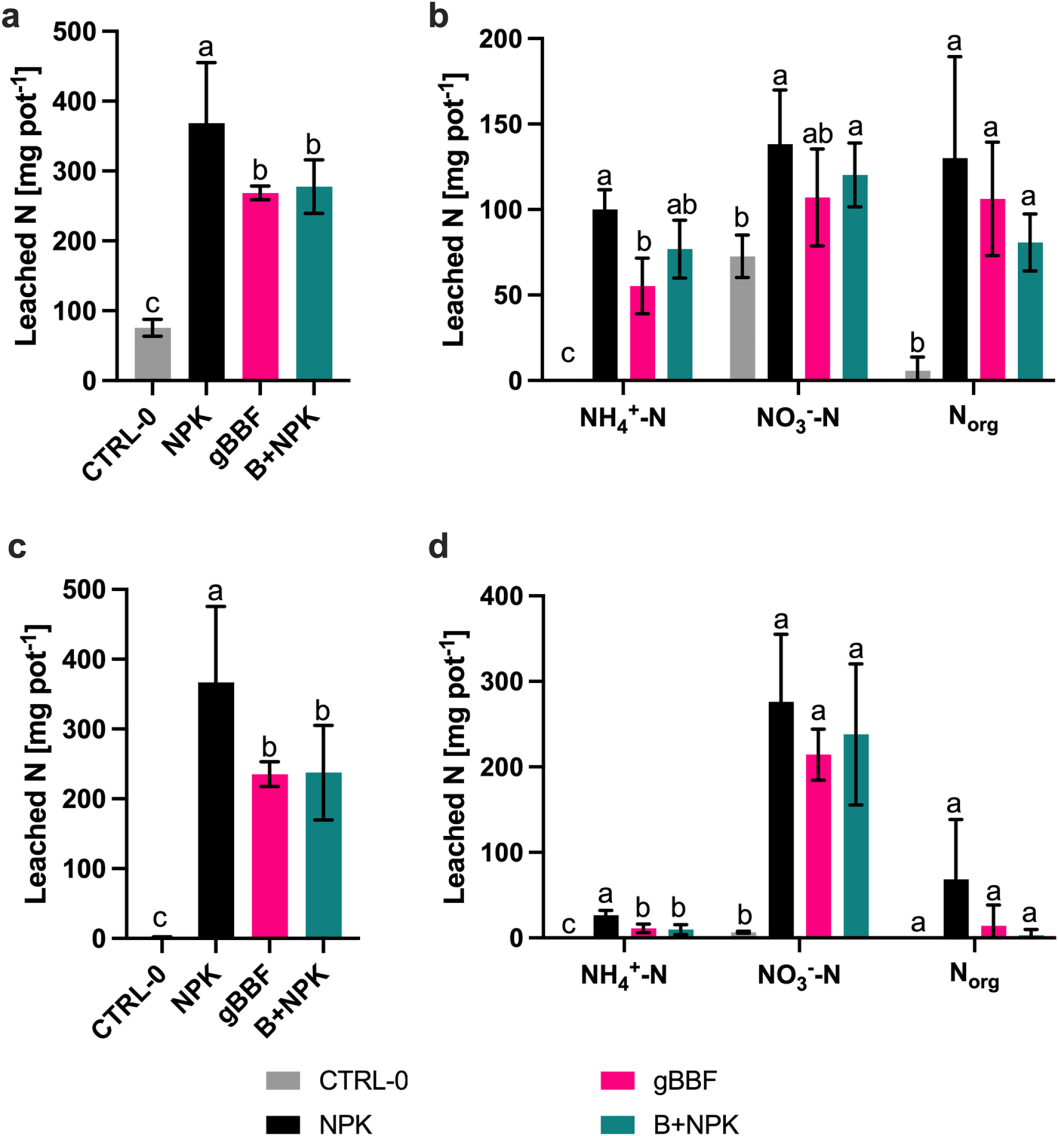
Total nitrogen (N) and individual N fractions leached from the pots during a leaching event after 35 days (**a**, **b**) and 63 days (**c**, **d**) with 30 L m^−2^ each. Different N fractions are presented as ammonium–N, nitrate–N and organic N (NH_4_^+^-N, NO_3_^−^-N and N_org_, respectively). Data are presented as means ± standard deviation (n = 5). CTRL-0: non-fertilized control. NPK: granulated mineral fertilizer. gBBF: granulated biochar-based NPK fertilizer. B + NPK: co-application of non-granulated biochar and granulated NPK fertilizer. Different letters above error bars indicate significant differences between the treatments within each N fraction (one-way analysis of variance, *p* < 0.05, Tukey’s post-hoc test).

When the first leaching event was performed, at 35 days after sowing, the cabbage plants had developed to the sixth unfold foliage (BBCH 16^27^), and at the time of the second leaching event, at 63 days after sowing, cabbage head formation had started (BBCH 41^27^). The biochar amendment significantly reduced total N losses in both individual leaching events in the range of 26–35%, compared to NPK, independent of the biochar application method (Fig. 4a,c). Cumulative TN losses were significantly reduced with gBBF and B + NPK compared to NPK by 31% and 30%, respectively (*p* < 0.05, Tables 5 and 6). TN losses summed up to 15% of initially fertilized N for gBBF and B + NPK and to 21% for NPK. In the non-fertilized pots, all the native mineralized N from the soil was leached during the first leaching event; no N remained to be leached at the second leaching (Fig. 4). NH_4_^+^-N loss was significantly reduced by 45% (*p* < 0.05) with gBBF and by 23% (*p* = 0.10) with B + NPK during the first leaching event compared to the NPK treatment (Fig. 4b). In the second leaching event, the NH_4_^+^-N losses were significantly decreased by 58% and 63% with gBBF and B + NPK (*p* < 0.05), respectively, with lower absolute NH_4_^+^-N losses for all treatments compared to the first leaching event (Fig. 4d). Cumulative NH_4_^+^-N leaching was reduced by 49% with gBBF and by 35% with B + NPK compared to NPK (*p* < 0.05, Tables 5 and 6) and made up 13–17% of TN lost via leaching. Therefore, NH_4_^+^-N retention was less critical than leaching of other N species, most importantly nitrate. NO_3_^-^-N leaching was reduced with gBBF and B + NPK in both individual leaching events compared to NPK fertilized pots, but not statistically significant (Fig. 4b,d, Tables 5 and 6). Leaching of organic N (N_org_) was reduced with gBBF and B + NPK compared to NPK during both individual leaching events (Fig. 4b,d) and statistically significant for the cumulative N_org_ loss for B + NPK compared to NPK (− 59%, *p* < 0.05, Table 5). In general, the leaching of N_org_ was higher in the first leaching event as compared to the second.

**Table 5:** Cumulative leaching of nitrogen (N) fractions during leaching events (in mg N pot^−1^): total dissolved N (TN), nitrate-N (NO_3_^−^-N), ammonium-N (NH_4_^+^-N) and organic N (N_org_). Data are presented as means ± standard deviation (n = 5). Different letters show significant differences between the treatments within each N fraction (one-way analysis of variance, p < 0.05, Tukey’s post-hoc test). LOQ: limit of quantification. CTRL-0: non-fertilized control. NPK: granulated mineral fertilizer. gBBF: granulated biochar-based NPK fertilizer. B+NPK: co-application of non-granulated biochar and granulated NPK fertilizer.

Treatment	TN	NO_3_^−^–N	NH_4_^+^–N	N_org_
CTRL-0	85 ± 12 c	79 ± 13 b	< LOQ	6 ± 8 c
NPK	736 ± 194 a	415 ± 98 a	127 ± 12 a	199 ± 121 a
gBBF	504 ± 22 b	322 ± 52 a	65 ± 18 b	123 ± 46 ab
B + NPK	516 ± 102 b	358 ± 89 a	86 ± 14 b	81 ± 18 b

**Table 6:** Statistical results of one-way analyzes of variance of the cumulative nitrogen (N) leaching losses from two leaching events for total dissolved N and each individual N fraction for the factor ‘fertilizer type’ (NPK, gBBF, B + NPK, excluding the non-fertilized control (CTRL-0)).

Cumulative Leaching	Total N		Ammonium		Nitrate		N_org_	
Factor	F	p	F	p	F	p	F	p
Fertilizer type	7.62	**0.014**	19.18	**< 0.001**	1.55	0.269	4.38	0.052
Block	2.36	0.140	0.63	0.655	0.86	0.526	2.40	0.136

Additionally, the block effect is presented ‘Block’. N_org_: organic N.

Significant values are displayed in bold.

**Figure 5:**
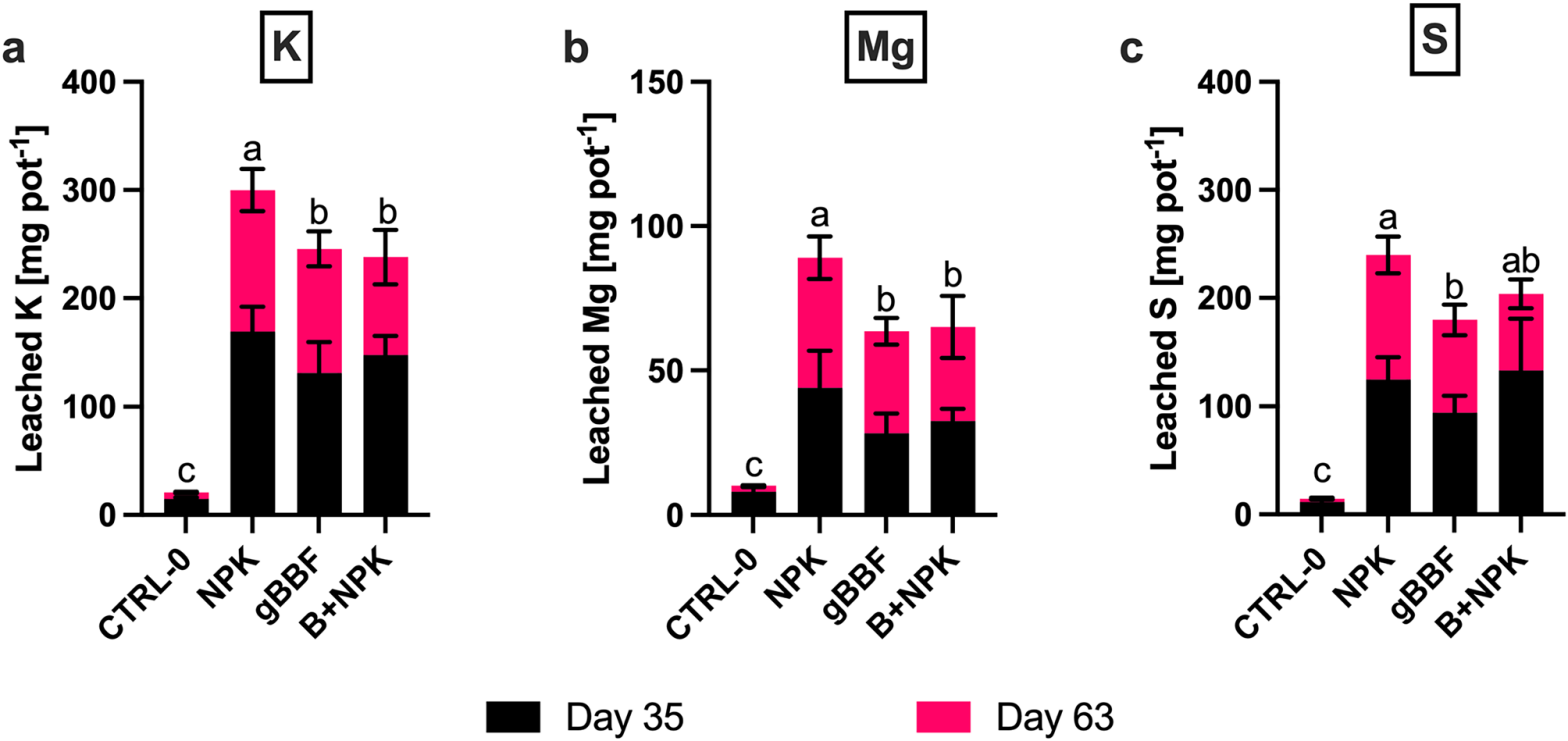
Amount of (**a**) potassium (K), (**b**) magnesium (Mg) and (**c**) sulfur (S) leached from cabbage pots as the result of two leaching events (each 30 L m^−2^ precipitation) at 35 days and 63 days after sowing. CTRL-0: non-fertilized control. NPK: granulated mineral fertilizer. gBBF: granulated biochar-based NPK fertilizer. B + NPK: co-application of non-granulated biochar and granulated NPK fertilizer. Data are presented as means ± standard deviation (n = 5). Different letters above error bars indicate a significant difference in cumulative nutrient loss between the different treatments (one-way analysis of variance, *p* < 0.05, Tukey’s post hoc test).

Cumulatively leached amounts of K ranged between 13 and 15% of the fertilized K. Mg leaching summed up to 20–30% of fertilized Mg, and S leaching was between 18 and 23% of fertilized S in the different treatments. Cumulative K leaching was significantly reduced by 21% with B + NPK and by 18% with gBBF compared to the NPK treatment (*p* < 0.05 Fig. 5a, Table 7). Cumulative Mg leaching loss was significantly reduced with gBBF by 28% and by 27% with B + NPK compared to NPK (*p* < 0.05, Fig. 5b, Table 7). Further, gBBF significantly reduced Mg leaching by 36% compared to NPK during the first leaching event (*p* < 0.05), while B + NPK only reduced it by 25% (*p* > 0.05, Fig. 5b). Sulfur leaching was consistently reduced with gBBF during both leaching events compared to NPK, summing up to a cumulative reduction by 25% (*p* < 0.05, Fig. 5c). The B + NPK treatment reduced S leaching by 38% in the second leaching event compared to NPK (*p* < 0.001), but the cumulative S loss was not significantly reduced compared to NPK (Fig. 5c). Phosphorus leaching was not affected by the different treatments during both leaching events and ranged between 0.3 and 0.8 mg P pot^−1^ (Table S8). Leachate volumes were not affected by biochar addition within the fertilized treatments (Table S8).

**Table 7:** Statistical results of one-way analyzes of variance of the cumulative potassium (K), magnesium (Mg) and sulfur (S) leaching loss from two leaching events for the factor ‘fertilizer type’ (NPK, gBBF, B + NPK, excluding the non-fertilized control (CTRL-0)).

Cumulative Leaching	K		Mg		S	
Factor	F	*p*	F	*p*	F	*p*
Fertilizer type	4.948	**0.040**	6.75	**0.019**	6.97	**0.020**
Block	0.6838	0.623	2.07	0.177	0.56	0.697

Additionally, the block effect is presented ‘Block’.

Significant values are displayed in bold.

### Soil N content, N balance, and soil pH

**Figure 6:**
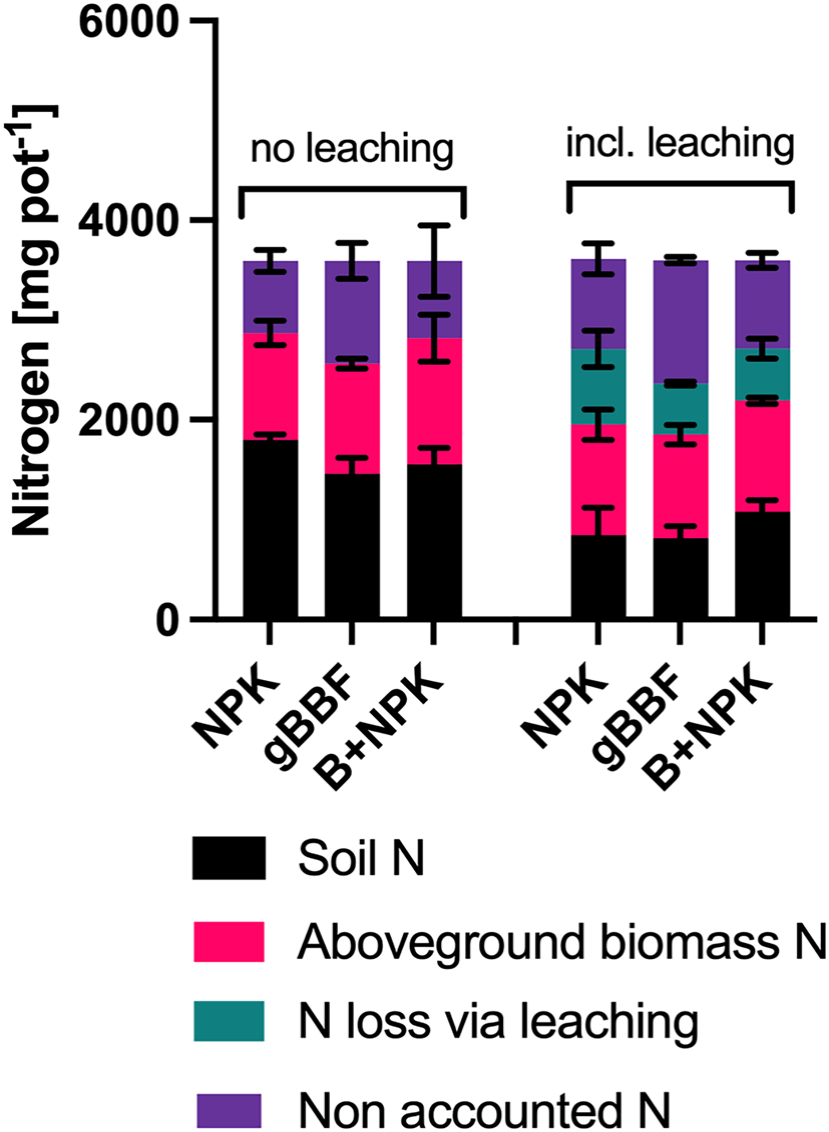
Nitrogen (N) balance for all fertilized treatments: residual total N in the soil after harvest, based on soil extractions with 0.01M CaCl_2_, total N quantified in aboveground biomass, total N lost via leaching and non-quantified N. Non accounted N was calculated as difference between the total fertilizer addition to the soil (including native mineralized soil-N at the start of the experiment) and the sum of the other N pools. NPK: granulated mineral fertilizer. gBBF: granulated biochar-based NPK fertilizer. B + NPK: co-application of non-granulated biochar and granulated NPK fertilizer to the soil. Data are presented as means ± standard deviation (n = 5).

Extractable N fractions from the soil after 36 and 70 days, i.e., shortly after the nutrient leaching events, were, when cumulated over all N fractions, not significantly changed by the different amendments (Fig. S19). Still, extractable NH_4_^+^-N contents were significantly lower for gBBF and B + NPK compared to NPK after 36 days and for B + NPK also after 70 days, but absolute NH_4_^+^–N contents were in general lower compared to NO_3_^−^–N and N_org_ (Fig. S19). Soil urease activity was not affected by the different fertilizer amendments based on samples taken after 70 days and at the harvest on day 116 (Fig. S18). After harvest, a considerable amount of 0.01 M CaCl_2_ extractable N remained in the soil in all treatments except in the leached non-fertilized control (Table S9). Treatments that included leaching events had between 25 and 55% lower residual extractable soil N contents compared to the treatments without leaching (Fig. 6 and Table S9). Virtually all N that remained in the soil was present as NO_3_^-^-N and ranged between 290 to 390 mg kg^−1^ for fertilized treatments without leaching events and from 180 to 200 mg kg^−1^ for fertilized treatments with leaching events (Table S9). In the treatments without leaching, NPK fertilized control pots had significantly higher extractable NO_3_^-^-N contents compared to gBBF and B + NPK (*p* < 0.05, Fig. 6 and Table S9). Non-accounted N ranged between 20 and 35% of total N in the pots (fertilizer + native extractable, mineral soil N, Fig. 6). The non-accounted N is assumed to be the sum of N in (1) below-ground biomass (roots were not analyzed for N content), (2) N emitted during the pot trial as NH_3_, N_2_O, or N_2_, (3) soil N that was non-extractable with 0.01 M CaCl_2_ and (4) N attached to leached particles not quantified due to leachate filtration to < 0.45 µm before analysis.

The leaching events contributed to a significant pH change in the NPK treatment from 7.6 in the non-leached soils to 8.0 in the leached soils (*p* < 0.05, Table S9). For gBBF and B + NPK, soil pH did not increase significantly during the trial after leaching and ranged between 7.7 and 7.8 (Table S9).

### Impact of soil incubation on N content and porosity of gBBF

**Figure 7:**
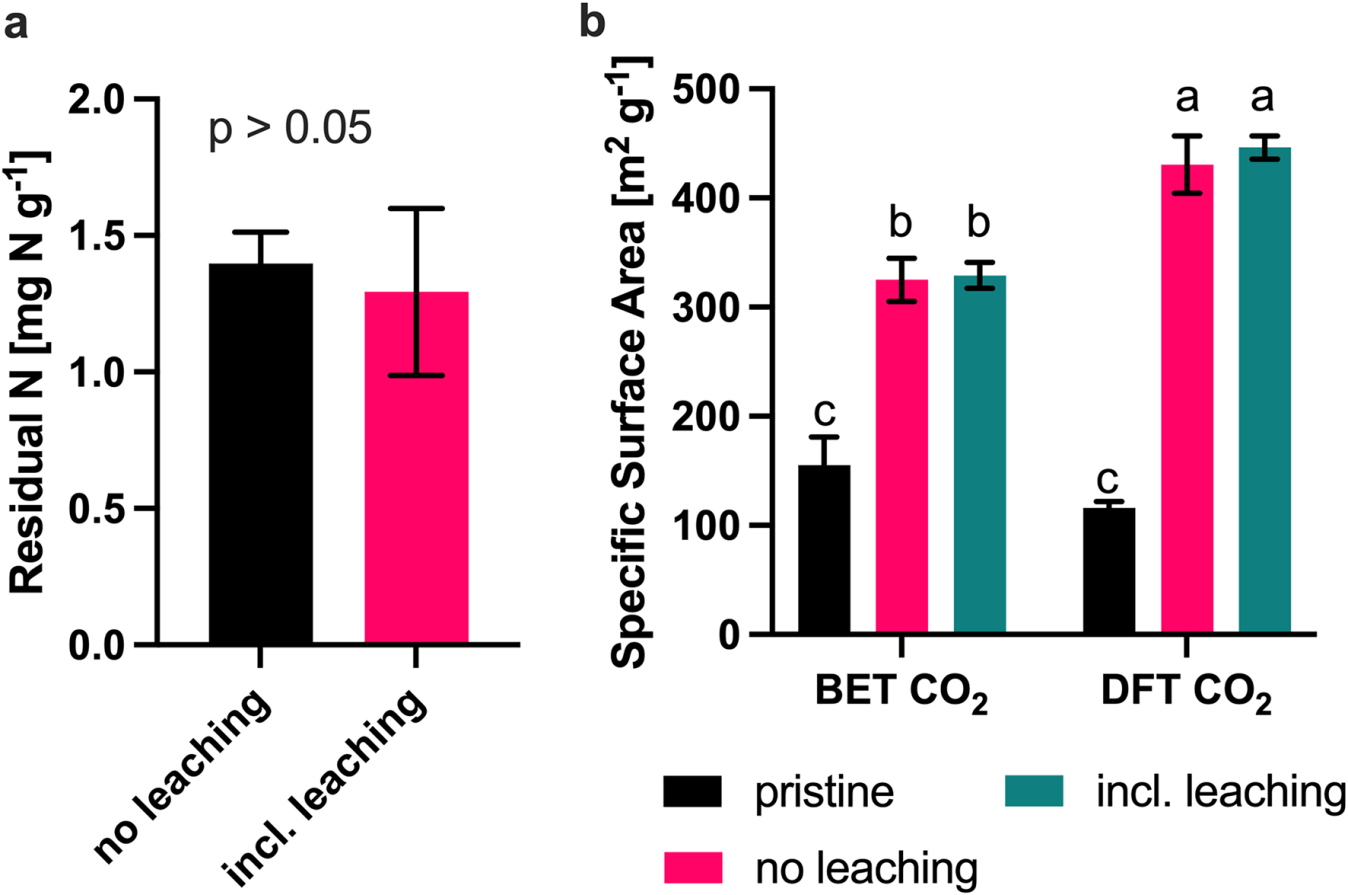
(**a**) Residual nitrogen (N) extracted with 2 M KCl solution for 2 h from granulated biochar-based fertilizer (gBBF) that was sampled from the soil after the harvest of white cabbage plants. (**b**) The specific surface area of pristine gBBF and soil-incubated gBBF was calculated based on CO_2_ adsorption isotherms according to the Brunauer-Emmet-Teller (BET) model or Density Functional Theory (DFT). Samples were analyzed both from the pots without or including two leaching events. Data are presented as means ± standard deviation (n = 5 in panel a and n = 3 in panel (**b**)). Different letters above error bars indicate a significant difference between the different treatments at *p* < 0.05 (one way analysis of variance, *p* < 0.05, Tukey’s post hoc test).

Low residual amounts of N were quantified in gBBF granules sampled from the soil after harvest in the pots treated with or without the conduction of leaching events (1.3–1.4 mg g^−1^; i.e., 1.5% of the initial N content, Fig. 7a). Thus, almost all N originally contained in the gBBF fertilizer (87 mg g^−1^) was released to the soil during the greenhouse trial. Still, the extractable N content in gBBF granules were approximately six times higher compared to the CaCl_2_-extractable N from the bulk soil in the respective treatments (0.2–0.3 mg g^−1^, Table S9).

The gBBF granules sampled from the soil after harvest had a significantly higher SSA based on CO_2_ adsorption compared to the original, non-incubated gBBF samples, independent of whether leaching events were applied or not (*p* < 0.001, Fig. 7b). The soil-aged gBBF granules had a SSA in the range of 300–360 m^2^ g^−1^ (BET method) and 400–480 m^2^ g^−1^ (DFT method) based on CO_2_ adsorption. This is in the same range of SSA measured for the pristine non-granulated biochar (358 m^2^ g^−1^ (BET) and 516 m^2^ g^−1^ (DFT), Table 2). The adsorption isotherms recorded for the soil-aged gBBF were well aligned to the isotherm recorded for the pristine, non-granulated biochar, which was also the case for the pore size distributions (Figs. S21 and S22).

## Discussion

The plant growth study confirmed the potential of granulated BBFs to maintain crop productivity while improving soil nutrient retention compared to mineral fertilization alone. Still, the experiments indicated better plant growth in terms of aboveground biomass yield and nutrient uptake in the presence of non-granulated biochar co-applied with fertilizer. Three different mechanisms might be relevant for this observation. First, under optimized soil moisture (constant 65% WHC, no leaching event), the gBBF decreased the uptake of important macronutrients such as P, Mg, and S compared to the co-application of biochar with fertilizer, indicating immobilization of these nutrients in the gBBF potentially due to the closer contact of nutrients and biochar in the gBBF and subsequent higher nutrient adsorption on biochar surfaces. Secondly, in line with the literature^17^, combined granulation of biochar and fertilizer significantly decreased the porosity of the biochar contained in the gBBF, which could be due to compression of the individual biochar particles by granulation and biochar pore blockage by the fertilizer, as indicated by gas adsorption, SEM and EDX results. The reduction in biochar porosity might negatively impact the otherwise positive effects of pure biochar (co-applied with NPK) on soil properties and plant growth. However, our results also confirmed that the biochar in the gBBF regained its original microporosity during soil incubation, which we explain by the dissolution of nutrients in soil pore water. Thirdly, with the gBBF, individual biochar particles were less uniformly distributed in the soil compared to co-applied biochar, which might limit the potential of positive biochar impacts on plant-soil interactions.

Improved crop growth with leaching in the biochar treatments was not consistently linked to higher nutrient uptake, even though improved soil nutrient retention was observed with biochar amendments compared to pure mineral fertilization, most likely due to the high fertilization level used in the experiment. Under a more limited fertilization scheme, biochar-amended plants might have yielded higher cabbage yields compared to sole mineral fertilization since the reduced nutrient leaching with biochar would have had a more pronounced positive effect on crop growth. The still higher biomass production with biochar in the leaching treatments might be explained by the stabilization of soil pH close to a range more favorable for cabbage growth (i.e., a pH between 6.0 and 7.5^28^) while soil pH rose to 8.0 with sole NPK fertilization. The soil pH measurements indicated that biochar retained more protons in the soil, i.e., derived from organic acids in root exudates or from the mineral fertilizer (which had a slightly acidic pH).

Our initial hypothesis, that combined granulation of biochar and fertilizer would improve biochar’s nutrient retention effectivity in soil, was valid for NH_4_^+^-N leaching during the first leaching event. However, total N losses were not impacted by biochar pretreatment during both leaching events. The hypothesis was further valid for Mg and S leaching during the first leaching event. However, cumulative reductions in nutrient leaching were reduced to the same extent for both biochar application methods compared to sole NPK fertilization. It is therefore suggested that although there is an initial improvement in the retention of some nutrients with the gBBF, this difference between gBBF and biochar-co-application with fertilizer will even out over time likely due to the progressing nutrient release from the gBBF largely restoring initial biochar properties, even despite the additional milling to < 1 mm. Nonetheless, biochar properties will change over time by aging in soil^29,30^, but this requires considerably longer periods of time than in the present study.

In the present study, lower nutrient losses from soil amended with biochar were observed. This is likely due to the interaction of fertilizer components with the biochar surface since leachate volumes were not affected by the treatments. The fact that the biochar used here was more microporous than mesoporous indicates a relevant role of biochar micropores to improve soil nutrient retention. However, we did not quantify biochar macroporosity (e.g., by mercury intrusion), which might also contribute to interaction with dissolved nutrients.

Nitrogen was applied to the pots exclusively as urea, which, in a first step, is mineralized in soil to NH_4_^+^-N by urease enzymes^31^. Urea-N was better retained in the gBBF during the extraction and incubation experiments compared to the loose mixture of B + NPK, which is in line with literature^15,16^, likely due to improved electrostatic interactions between the NH_2_ groups in urea and negatively charged surface sites of the biochar. Urea molecules trapped in biochar pores in gBBF were potentially less available for ammonification until they were diffused out of the biochar pores to the soil, which was also partly indicated by less extractable NH_4_^+^-N from the gBBF-amended soil samples taken 36 days after the beginning of the pot trial. Still, since the first leaching event in the greenhouse experiment was conducted after 35 days, most of the urea might have already been released and mineralized from the gBBF granule as indicated by the incubation experiments, where after 9 days, almost all N was released to the incubation liquid. If the leaching events had occurred at an earlier stage of the experiment, the reduction in N leaching provided by gBBF may have been more pronounced. Still, once urea was released from gBBF, it was mineralized closer to the biochar matrix compared to B + NPK, which may have eased NH_4_^+^-N adsorption on biochar surfaces with the gBBF^32^ and can explain the lower leaching of NH_4_^+^-N with gBBF compared to B + NPK in the first leaching event. Adsorption of NH_4_^+^-N and urea-N on the biochar surface might be attributed to negatively charged carboxyl and phenolic groups^32,33^ on biochar, which can also explain the general lower leaching loss of K^+^ and Mg^2+^ in the treatments with biochar amendments in line with the literature^34,35^. Dissolved cations might also be retained in biochar pores by interaction with OH–$\pi$-bound water or water bound by Van-der-Waal force at the biochar surface^36^. The improved retention of anions like nitrate (NO_3_^−^) and sulfur, most likely present as sulfates (SO_4_^2−^), may be attributed to positively charged biochar surface sites, such as O^+^-heteroatoms in aromatic rings that can form during pyrolysis above 700 °C, which applies to the biochar used in our study^33,37^. Still, non-modified and non-aged biochars have only low nitrate adsorption and anion exchange capacities, which might be the reason why NO_3_^−^-N leaching only tended to be reduced with biochar amendments in the present study compared to the control. Nitrate retention in biochar has been shown to rather evolve during soil aging of biochar surfaces and interaction with organic soil amendments or potentially organic acids derived from root exudates^30,32,33,38–41^. The lower extractable soil NO_3_^−^-N content in gBBF and B + NPK treatments compared to NPK in the absence of leaching after harvest might indicate that at the end of the experiment, some NO_3_^−^-N was captured by biochar that was not extractable with 0.0125 M CaCl_2_.

We observed a reduction in cumulative N leaching by 32% with the gBBF compared to NPK. Three earlier studies found either no reduction in N leaching^17^, reductions by 44–61%^9^, or 6–9%^42^ with the application of gBBFs compared to fertilization without biochar. The observation that different studies on gBBFs found contrasting results on relative changes in N leaching losses may be explained by (1) an interaction of N leaching from gBBFs and soil type and (2) the gBBF characteristics (e.g., N speciation, biochar type, biochar to N ratio, and additive content). We prepared a gBBF using only trace amounts of a binding agent to be able to solely elucidate the impact of biochar itself in the gBBF on nutrient leaching. In earlier studies, additives like bentonite or paraffin wax were added to the gBBFs at significantly higher quantities, not just traces (e.g. ~ 50% w/w), which might have affected their agronomic impact e.g., on N leaching^7–9,15–17^. Furthermore, all these studies only included a fertilizer-only control, but no control that included the additives, or biochar concomitant to the fertilizer. Therefore, the observed differences in N leaching between different studies might be linked to the additives used in the different BBFs.

The heavy precipitation events conducted during the present study of 30 mm within 1 h reflect events that will likely occur more often during crop cultivation periods in temperate climates in the future with progressing climate change^43,44^. The reduction in nutrient leaching provided by the gBBF is promising as less environmental impact per unit of produced crop could be achieved. However, the additional incorporation of biochar into the granulated fertilizer at a rate of 65% (w/w) would increase fertilizer material costs by 930–1850 € per ton of mineral fertilizer, assuming a biochar cost of 500–1000 € t^−1^; it would also require additional fuel for field application of the same amounts of nutrients, since more mass has to be transported to and on the field. Further, additional costs might occur during the production of the gBBF, e.g., due to biochar milling, which would have to be addressed in follow-up studies. At the same time, the CO_2_ sequestration potential of 6.3 t CO_2_ per ton of mineral fertilizer that is achieved by a gBBF as used here would translate to an income of 1000 € for the producer of the biochar-based fertilizer by CDR trading/carbon sink service revenues, assuming a biochar price index^45^ of 180 $ (t CO_2_)^−1^.

## Conclusion

In the sandy soil used in the present study, biochar amendment had no statistically significant benefit on biomass yields under optimal growing conditions. With heavy rainfall (leaching) events, biochar increased yields significantly when co-applied with NPK fertilizer. Nutrient uptake of several macronutrients was significantly lower with gBBF compared to co-application of biochar and fertilizer in absence of leaching events, indicating immobilization in gBBF. In contrast to our hypothesis, granulation of biochar with mineral fertilizers did not significantly alter biochar effects on nutrient leaching compared to the co-application of non-granulated biochar with mineral fertilizer. Leaching events reduced yields across all treatments, however, biochar amendment counteracted the negative effect to some degree, especially with co-application of biochar, by improving plant nutrition with macronutrients. Since the granulated BBF provided similar crop yields compared to standard mineral fertilization, the combination of biochar and fertilizer via granulation can be recommended to enable biochar use with standard agricultural machinery. Further, biochar application provided an improved ratio of environmental effects (in particular reduced leaching) per unit of crop produced. Our study highlighted that granulated BBF can easily be produced without large amounts of various (binding) additives, as done in some earlier studies. A limitation of the current study is that the experiments included only one type of biochar, soil and crop and further, the results for crop growth and nutrient leaching might be different in the field due to the different environmental conditions as compared to the greenhouse. Future studies should investigate and optimize the effects of different biochar types and particle sizes, biochar-to-nutrient ratios, nutrient speciation, and production techniques of granulated or pelleted BBFs on plant growth, nutrient retention, and soil-borne greenhouse gas emissions in different soils (since reductions in N_2_O emissions are a common finding with biochar use^5^) both in greenhouse and field trials. Combined granulation of biochar and fertilizer is a way to ease adopting biochar application in agriculture to convey the positive environmental effects of biochar application to soils on a broader scale.

## Supplementary Information

Supplementary Information.

## Data Availability

The original data of this study is available on Zenodo with the following link: 10.5281/zenodo.12098693.
