# Publisher Correction: Granulation compared to co-application of biochar plus mineral fertilizer and its impacts on crop growth and nutrient leaching

**DOI:** 10.1038/s41598-025-87051-2

**Published:** 2025-01-30

**Authors:** Jannis Grafmüller, Jens Möllmer, E. Marie Muehe, Claudia I. Kammann, Daniel Kray, Hans-Peter Schmidt, Nikolas Hagemann

**Affiliations:** 1 Institute of Sustainable Energy Systems (INES), Offenburg University of Applied Sciences, Offenburg, Germany; 2 Ithaka Institute, Arbaz, Switzerland; 3 Ithaka Institute, Goldbach, Germany; 4 Plant Biogeochemistry, Department of Geosciences, University of Tübingen, Tübingen, Germany; 5 Environmental Analytics, Agroscope, Zurich, Switzerland; 6 Institut für Nichtklassische Chemie e.V. (INC), Leipzig, Germany; 7 Plant Biogeochemistry, Department of Applied Microbial Ecology, Helmholtz Centre for Environmental Research-UFZ, Leipzig, Germany; 8 Department of Applied Ecology, Hochschule Geisenheim University, Geisenheim, Germany

Correction to: *Scientific Reports* 10.1038/s41598-024-66992-0, published online 17 July 2024

The original version of this Article contained errors in Table 6, where the “Fertilizer type” and “Block” for the Cumulative Leaching were incorrectly given as “NPK” and “gBBF”. The correct and incorrect values appear below.

Incorrect:

**Table 6:** Statistical results of one-way analyzes of variance of the cumulative nitrogen (N) leaching losses from two leaching events for total dissolved N and each individual N fraction for the factor ‘fertilizer type’ (NPK, gBBF, B + NPK, excluding the non-fertilized control (CTRL-0)).

Cumulative Leaching	Total N		Ammonium		Nitrate		N_org_	
Factor	F	p	F	p	F	p	F	p
NPK	7.62	**0.014**	19.18	**< 0.001**	1.55	0.269	4.38	0.052
gBBF	2.36	0.140	0.63	0.655	0.86	0.526	2.40	0.136

Additionally, the block effect is presented ‘Block’. N_org_: organic N.

Significant values are displayed in bold.

Correct:

**Table 6:** Statistical results of one-way analyzes of variance of the cumulative nitrogen (N) leaching losses from two leaching events for total dissolved N and each individual N fraction for the factor ‘fertilizer type’ (NPK, gBBF, B + NPK, excluding the non-fertilized control (CTRL-0)).

Cumulative Leaching	Total N		Ammonium		Nitrate		N_org_	
Factor	F	p	F	p	F	p	F	p
Fertilizer type	7.62	**0.014**	19.18	**< 0.001**	1.55	0.269	4.38	0.052
Block	2.36	0.140	0.63	0.655	0.86	0.526	2.40	0.136

Additionally, the block effect is presented ‘Block’. N_org_: organic N.

Significant values are displayed in bold.

The original Article has been corrected.

