# Supplementary material for: Granulation compared to co-application of biochar plus mineral fertilizer and its impacts on crop growth and nutrient leaching
Source: Sci Rep. 2024 Jul 17;14:16555. doi: 10.1038/s41598-024-66992-0 (PMC11255204; doi:10.1038/s41598-024-66992-0)
Supplement: Supplementary file 1 — Supplementary Information. [file 41598_2024_66992_MOESM1_ESM.pdf]

Granulation compared to co-application of biochar plus mineral fertilizer and its impacts on crop growth and nutrient leaching.

Jannis Grafmüller[1,2,3,4,*], Jens Möllmer[5], E. Marie Muehe[3,6], Claudia I. Kammann[7], Daniel Kray[1], Hans-Peter Schmidt[2] and Nikolas Hagemann[2,4]

[1]Institute of Sustainable Energy Systems (INES), Offenburg University of Applied Sciences, Germany

[2]Ithaka Institute, Arbaz (Switzerland) and Goldbach (Germany)

[3]Plant Biogeochemistry, Department of Geosciences, University of Tübingen, Tübingen, Germany

[4]Environmental Analytics, Agroscope, Zurich, Switzerland

[5]Institut für Nichtklassische Chemie e.V. (INC), Leipzig, Germany

[6]Plant Biogeochemistry, Department of Applied Microbial Ecology, Helmholtz Centre for Environmental Research - UFZ, Leipzig, Germany

[7]Department of Applied Ecology, Hochschule Geisenheim University, Geisenheim, Germany

*Corresponding author: Jannis Grafmüller (jannis.grafmueller@hs-offenburg.de)

Supplementary material

1. Supplementary information on material and methods

1.1. Gas adsorption of biochar and fertilizer

CO_2 adsorption isotherms were recorded at 273.15 K (cryoTune 195, 3P Instruments GmbH& Co. KG, Odelzhausen, Germany) in a relative pressure range of 3 x 10^{-5} to 3 x 10^{-2} and at 77 K for N_2 isotherms in a relative pressure range of 1.2 x 10^{-3} to 9.9 x 10^{-1}. Samples were outgassed at 30 °C[1] under vacuum for a minimum of 12 hours. At this temperature, outgassing mass losses were in the range of the water contents of the samples measured with Karl Fischer titration (see Table S1 for further details) and no precipitated salt was visible at the top of the measurement cell in case of NPK and gBBF, which was the case when outgassed at e.g., 90 °C.

1.2. Scanning Electron Microscopy (SEM)

For scanning electron microscopy (SEM), a gBBF granule and its cross section (after preparation with a scalpel) were mounted on an aluminum stub using adhesive carbon tape and sputtered with gold on the outer surface. Secondary electron imaging was performed in a Jeol JSM-6610LV (Joel Ltd., Tokyo, Japan). Electron dispersive X-ray (EDX) mapping was done with a 10 minute measurement time, 10 kV acceleration voltage, and a 20 mm^2 silicon drift detector (X-Max, Oxford Instruments, Oxford, United Kingdom).

1.3. N-release characteristic of fertilizers

Repeated extractions

For the repeated extractions, samples were weighted into 100 mL Erlenmeyer flasks (equivalent of 0.4 g of N per flask), 50 mL of a 0.0125 M $CaCl_2$ solution were added and shaken at 125 rpm on a horizontal shaker for 1h. Subsequently, the extracts were filtered through a filter paper. The filter paper including the decanted solid fractions of biochar and fertilizer was returned into the sample flask with another 50 mL of fresh extractant. The procedure was repeated twice, with two hours and 45 hours extraction times, respectively, summing up to a total extraction time of 48h. Liquid extracts were filtered to <0.45 µm (Chromafil PES Xtra 45/25, Machery-Nagel, Düren, Germany) and analyzed for total nitrogen (TN) content.

Extracted TN from the sample during each extraction step was calculated as follows,

$$N_{extracted,i} = \frac{c_{N,i} \cdot \left(V_{E,i} + V_{ER,i-1}\right) - c_{N,i-1} \cdot V_{ER,i-1}}{m_S \cdot ß_N} \cdot 100\%$$

where

- $N_{extracted,i}$ is the mass fraction of extracted N during extraction step *i* in relation to TN initially contained in the solid sample (in %),
- $c_{N,i}$ is the TN concentration in the filtered extract from extraction step *i*,
- $V_{E,i}$ is the volume of extractant added to the sample for extraction step *i,*
- $V_{ER,i-1}$ is the remaining volume of extractant in the flask from the extraction step before,
- $c_{N,i-1}$ is the TN concentration in the extractant from extraction step before, m_S is the mass of the N containing solid fertilizer and
- $ß_N$ is the TN content in the solid fertilizer

Incubation experiment

Released TN during the incubation experiment was calculated as follows:

$$N_{released,i} = \frac{c_{N,i} \cdot V_{tot}}{m \cdot \beta_N \cdot (V_{tot} - i \cdot V_W)}$$

where

- $N_{released,i}$ is the mass fraction of extracted TN after sampling *i* in relation to the initial total N contained in the solid sample (in %),
- $c_{N,i}$ is the TN concentration in the filtered extract from sampling *i*,
- V_{tot} *is the total incubation volume (=100 mL)*
- **m** is the mass of the fertilizer weighed into the bottle
- $ß_N$ is the TN content in the fertilizer (w/w)
- V_W is the volume of de-ionized water added to the bottle after sampling (=5mL),
- ***i*** is the number of total samples taken, starting with i=0.

1.4. Greenhouse trial maintenance

The pots were arranged in a randomized block design on a greenhouse table with east-west orientation (located at 48°29'01.7"N 7°56'25.9"E) with an average temperature of 22±6 °C, an average humidity of 71±24 % and a CO_2 concentration of 498±51 ppm (Figure S2) and artificial lighting. In each pot, automated drip irrigation with 4 watering spots per pot was fixed (NetBow™, Netafim Ltd., Tel Aviv, Israel) which was operated daily with tap water. Once per week, the position of the pots was randomly re-arranged

within each block and pots were manually weighed, watered to 65% of the maximum water holding capacity (WHC, cf. Figure S3 for results), and the automated water dose was adjusted, if necessary. Maximum WHC were measured in triplicates in 1L pots after saturation of the individual soil-biochar and -fertilizer mixtures in a water bath for 16h and drainage of surplus water on a dry sand bed for 48h, with subsequent calculation of stored water mass per mass of dry matter mixture of soil, biochar and fertilizer. Two weeks after sowing, the seedlings were reduced to one plant per pot and an additional amount of 240 g soil (dry matter equivalent) was added to each pot on top to stabilize the remaining seedlings.

1.5. Biomass analysis

The dried and milled aboveground biomass (120 mg) was digested in a mixture of 3 mL 65 % HNO_3 and 2 mL 30 % H_2O_2 (both from Carl Roth, Karlsruhe, Germany) in a microwave (Mars 5 Xpress, CEM GmbH, Kamp-Lintfort, Germany) at 180 °C for 15 minutes (15 minutes ramp to 180 °C)[4]. Quantification of elements in the digests was performed with Inductively Coupled Plasma Optical Emission Spectroscopy (ICP-OES) in axial measurement configuration for all elements (icap 7000 series, Thermo Scientific, Waltham, USA). A multielement standard with manually added P and S standards was used for the calibration curves (Roti®Star standard solution IV and Single-element P solution, Carl Roth GmbH, Karslruhe Germany; Trace-Cert Sulfur standard, Sigma-Aldrich, St. Louis, USA). The digestion and ICP measurement were validated by digestion of a certified reference material (Table S6, ERM-CD281, European Commission DG JRC, Geel, Belgium).

1.6. Nitrogen analysis of fertilizer extracts, leachates, and soil extracts

$NH4^+$-N concentrations in the leachates, soil, and fertilizer extracts were quantified with a Berthelot reaction according to Rhine and colleagues[3] on 96 well microtiter plates. The calibration curve was obtained with NH_4Cl in the range of 0-20 mg NH_4^+-N L^{-1} and the absorbance was measured at 660nm in a microplate reader (Epoch2, Biotek Instruments, Winooski, USA). For NO_3-N quantification, a microplate reader method adapted from Hagemann et al. was applied[5]. A nitrate kit (Cat. No 1.09713, Merck KGaA, Darmstadt, Germany) was used and measurements were performed at 330 nm in a UV transparent 96-well plate (UV-Star®, Greiner Bio-One GmbH, Frickenhausen, Germany) using KNO_3 for the calibration in the range of 0 to 200 mg

NO_3^- L^{-1}. The microtiter plates were incubated for 60 minutes at 60 °C (ThermoMixer C, Eppendorf SE, Hamburg, Germany), after the reaction liquids from the kit were added to the sample.

TN in the leachates and extracts was quantified on a TOC-VCPN equipped with the total nitrogen measurement unit TNM-1 (Shimadzu Corporation, Kyoto, Japan). The calibration curve was obtained by a mixture of ammonium sulphate and potassium nitrate in the range of 0-100 mg N L^{-1} (N was present to 80% as NO_3-N and to 20% as NH_4-N in the standard).

2. Supplementary figures and tables

Table S1 Water content determined with Karl Fischer (KF) titration for the different samples and mass losses during outgassing at 30°C for 16 hours before gas adsorption measurements. gBBF: granulated biochar-based NPK fertilizer. NPK: granulated mineral NPK fertilizer. An outgassing temperature of 30 °C was chosen, since a higher outgassing temperature (90 °C) lead to salt precipitates at the top of the sample cell in case of the BBF and NPK samples, which would have disturbed the sample morphology before the adsorption experiments.

**Sample**	**Water content according to KF titration in % (w/w)**	**Mass loss during activation in % (w/w)**
Biochar	7.48 ± 0.05	8.4
gBBF	2.18 ± 0.02	2.9
NPK	2.41 ± 0.00	2.0

Table S2 *Main characteristics of the soil used in the greenhouse study. Concentrations are given based on dry matter, were applicable.*

**Parameter**	**Unit**	**Value**	**Method**
Soil organic matter	wt%	7.7	DIN ISO 10694: 1996-08
Total Organic Carbon	wt%	4.5	DIN ISO 10694: 1996-08
pH	1	7.4	VDLUFA Method book volume I, Chapter 5.1.1, 7th partial delivery, 2016
Electric Conductivity	μScm^{-1}	110	VDLUFA Method book volume I, Chapter 10.1.1, 1991
Salt content	$mg(100g)^{-1}$	58	VDLUFA Method book volume I, Chapter 10.1.1, 1991
Total Nitrogen	wt%	0.15	DIN ISO 13878:1998-11
NH_4-N	$mg(100g)^{-1}$	<0.05	VDLUFA Method book volume I, 3rd partial delivery, Chapter 6.1.4.1, 2002
NO_3-N	$mg(100g)^{-1}$	2.17	VDLUFA Method book volume I, 3rd partial delivery, Chapter 6.1.4.1, 2002
S_{min}	$mg(100g)^{-1}$	0.51	VDLUFA Method book volume I, A 6.3.1 (2016), Extraction with 0.0125 M $CaCl_2$
Potassium (K)	$mg(100g)^{-1}$	24.6	Calcium lactate extract, VDLUFA Method book volume I, 6th partial delivery, Chapter 6.2.1.1, 2012
Phosphorus (P)	$mg(100g)^{-1}$	13.0	Calcium lactate extract, VDLUFA Method book volume I, 6th partial delivery, Chapter 6.2.1.1, 2012
Magnesium (Mg)	$mg(100g)^{-1}$	8.8	Calcium chloride extract, VDLUFA Method book volume I, Chapter 6.2.4.1, 1991; ISO 22036:2008
Boron (B)	$mgkg^{-1}$	0.3	CAT extract, VDLUFA Method book volume I, 3rd partial delivery, Chapter 6.1.4.1, 2002
Manganese (Mn)	$mgkg^{-1}$	20	CAT extract, VDLUFA Method book volume I, 3rd partial delivery, Chapter 6.1.4.1, 2002
Copper (Cu)	$mgkg^{-1}$	4.6	CAT extract, VDLUFA Method book volume I, 3rd partial delivery, Chapter 6.1.4.1, 2002
Zinc (Zn)	$mgkg^{-1}$	9.5	CAT extract, VDLUFA Method book volume I, 3rd partial delivery, Chapter 6.1.4.1, 2002

Table S3 *Potential (pot.) and effective (eff.) cation exchange capacity of the soil used in the greenhouse study.*

**Parameter**	**Unit**	**Value**	**Method**
Cation exchange capacity (eff.)	$cmol^{+}kg^{-1}$	17.5	DIN EN ISO 11260:2011-09
Exchange acidity	$cmol^{+}kg^{-1}$	0.1	DIN EN ISO 11260:2011-09
Exchangable Mg (eff.)	$cmol^{+}kg^{-1}$	0.5	DIN EN ISO 11260:2011-09
Exchangable Ca (eff.)	$cmol^{+}kg^{-1}$	13.2	DIN EN ISO 11260:2011-09
Exchangable Na (eff.)	$cmol^{+}kg^{-1}$	<0.1	DIN EN ISO 11260:2011-09
Exchangable K (eff.)	$cmol^{+}kg^{-1}$	0.7	DIN EN ISO 11260:2011-09
Sum of exchangeable cations (eff.)	$cmol^{+}kg^{-1}$	14.4	DIN EN ISO 11260:2011-09
Cation exchange capacity (pot.)	$cmol^{+}kg^{-1}$	15.4	DIN ISO 13536: 1997-04
Exchangable Mg (pot.)	$cmol^{+}kg^{-1}$	0.5	DIN ISO 13536: 1997-04
Exchangable Ca (pot.)	$cmol^{+}kg^{-1}$	12.3	DIN ISO 13536: 1997-04
Exchangable Na (pot.)	$cmol^{+}kg^{-1}$	<0.1	DIN ISO 13536: 1997-04
Exchangable K (pot.)	$cmol^{+}kg^{-1}$	0.7	DIN ISO 13536: 1997-04
Sum of exchangeable cations (pot.)	$cmol^{+}kg^{-1}$	13.5	DIN ISO 13536: 1997-04

Table S4 *Particle size distribution and texture of the soil used for the greenhouse study.*

**Parameter**	**Unit**	**Value**	**Method**
Clay (<2µm)	wt%	11	DIN ISO 11277:2002:08
Coarse sand (0.63 - 2mm)	wt%	2	DIN ISO 11277:2002:08
Medium sand (0.2 - 0.63mm)	wt%	8	DIN ISO 11277:2002:08
Fine sand (0.063 - 0.2mm)	wt%	42	DIN ISO 11277:2002:08
Coarse silt (20-63 µm)	wt%	13	DIN ISO 11277:2002:08
Medium silt (6.3 - 20 µm)	wt%	14	DIN ISO 11277:2002:08
Fine silt	wt%	9	DIN ISO 11277:2002:08
Coarse soil (>2mm)	wt%	1	DIN ISO 11277:2002:08

Table S5 *Treatments prepared for the pot trial including the individual biochar application quantities and nutrient dosages. Biochar application quantities per hectare were calculated assuming a planting density of 40.000 cabbage plants ha^{-1} and a concentrated application of the biochar in the root-zone of the plants. n.a.: not applicable. Treatments with ID 4, 5, 6 and 7 received two leaching events for nutrient leaching at each 30 L m^{-2} precipitation after 35 days and 63 days of plant cultivation.*

**Treatment ID**	**Treatment**	**Leaching events**	**Biochar addition**			**Fertilized N[a]**	**Fertilized P[b]**	**Fertilized K[c]**	**Fertilized Mg[d]**	**Fertilized S[e]**
			**g pot^{-1}**	**wt%**	**t ha^{-1}**	**g pot^{-1}**				
1	NPK	0 L m^{-2}	n.a.	n.a.	n.a.	3.5	0.57	2.00	0.29	0.96
2	gBBF	0 L m^{-2}	26.7	0.63	1.07	3.5	0.60	2.13	0.28	1.08
3	B+NPK	0 L m^{-2}	26.7	0.63	1.07	3.5	0.57	2.06	0.31	0.96
4	CTRL-0	30 L m^{-2} (twice)	n.a.	n.a.	n.a.	n.a.	n.a.	n.a.	n.a.	n.a.
5	NPK	30 L m^{-2} (twice)	n.a.	n.a.	n.a.	3.5	0.57	2.01	0.29	0.96
6	gBBF	30 L m^{-2} (twice)	26.7	0.63	1.07	3.5	0.60	2.14	0.28	1.08
7	B+NPK	30 L m^{-2} (twice)	26.7	0.63	1.07	3.5	0.57	2.06	0.31	0.96

Table S6 *Recovery of phosphorus (P), potassium (K), magnesium (Mg), sulfur (S), calcium (Ca), copper (Cu), manganese (Mn) and zinc (Zn) from a certified reference material after microwave digestion and quantification with inductively coupled plasma optical emission spectroscopy (ICP-OES). The reference material was a rye grass listed as ERM-CD281 as sample no. 1010 by the European Commission. Errors indicate ± standard deviation of replicated (n=5) digestions.*

**Element**	**Quantified content in mg g^{-1}**	**Content according to analysis certificate in mg g^{-1}**	**Recovery in % (w/w)**
P	2.994 ± 0.064	2.8	106.9 ± 2.3
K	28.269 ± 0.455	34.0	83.1 ± 1.3
Mg	1.700 ± 0.026	1.6	106.3 ± 1.6
S	3.074 ± 0.059	3.4	90.4 ± 1.7
Ca	7.333 ± 0.130	6.3	116.4 ± 2.1
Cu	0.0102 ± 0.001	0.0102	99.5 ± 12.7
Mn	0.084 ± 0.001	0.082	102.7 ± 1.8
Zn	0.031 ± 0.002	0.0305	102.1 ± 7.9

Table S7 *Content of trace elements arsenic (As), lead (Pb), boron (B), cadmium (Cd), chromium (Cr), copper (Cu), manganese (Mn), nickel (Ni), mercury (Hg), silver (Ag) and zin (Zn) in the biochar and fertilizers. NPK: mineral nitrogen, phosphorus and potassium (NPK) fertilizer granule. gBBF: granulated biochar-based mineral NPK fertilizer.*

**Sample**	**As**	**Pb**	**B**	**Cd**	**Cr**	**Cu**	**Mn**	**Ni**	**Hg**	**Ag**	**Zn**
	**mg kg^{-1}**										
**Biochar**	<0.8	3	5	<0.2	13	2	208	9	<0.07	<5	43
**NPK**	<0.8	<2	2	<0.2	<1	2	10	<1	<0.07	<5	5
**gBBF**	<0.8	2	4	<0.2	7	2	140	6	<0.07	<5	30

Table S8 *Total volume of leachates and total amount of phosphorus (P) leached from the pots in the two precipitation events. Data is presented as mean ± standard deviation (n=5). Different letters within each column indicate a significant difference between the different treatments (Tukey's post-hoc test, p<0.05). CTRL-0: soil only - no fertilization, NPK: granulated, mineral nitrogen, phosphorus and potassium fertilizer. gBBF: granulated biochar-based NPK fertilizer. B+NPK: co-application of non-granulated biochar and granulated NPK fertilizer.*

**Treatment**	**Leachate amount in L (first precipitation)**	**Leachate amount in L (second precipitation)**	**Leached P in mg pot^{-1} (first precipitation)**	**Leached P in mg pot^{-1} (second precipitation)**
CTRL-0	0.459 ± 0.065 a	0.556 ± 0.025 a	0.21 ± 0.03 a	0.31 ± 0.04 a
NPK	0.442 ± 0.057 a	0.520 ± 0.069 ab	1.00 ± 0.18 a	0.78 ± 0.09 a
gBBF	0.425 ± 0.021 a	0.456 ± 0.016 b	0.99 ± 0.11 a	0.61 ± 0.13 a
B+NPK	0.438 ± 0.035 a	0.455 ± 0.025 b	1.08 ± 0.26 a	0.74 ± 0.14 a

Table S9 *Extractable nitrogen (N) species and pH in the soil samples taken from the pots after harvest. Extractable organic N (N_{org}), nitrate-N (NO3-N) and ammonium-N (NH4-N) was measured in filtered 0.0125M $CaCl_2$ extracts (1+4; m+V) and pH in the soil suspension with 0.0125M $CaCl_2$ (1+2.5; m+V). Data is presented as mean ± standard deviation (n=5). Different letters within a column indicate a significant difference between the treatments obtained from one-way analysis of variance ($p<0.05$, Tukey's post-hoc test). CTRL-0: soil only - no fertilization, NPK: granulated, mineral fertilizer. gBBF: granulated biochar-based NPK fertilizer. B+NPK: co-application of non-granulated biochar and granulated NPK fertilizer.*

**Treatment**	**Extractable N_{org}**	**Extractable $NO3^-$-N**	**Extractable $NH4^+$-N**	**pH**
	**mg $(100g)^{-1}$**	**mg $(100g)^{-1}$**	**mg $(100g)^{-1}$**	
**no leaching**				
NPK	3.6 ± 3.0 ab	38.9 ± 5.3 a	0.36 ± 0.01 a	7.6 ± 0.1 c
gBBF	5.0 ± 1.3 a	28.9 ± 3.7 b	0.32 ± 0.01 ab	7.7 ± 0.1 bc
B+NPK	6.4 ± 1.7 a	29.7 ± 5.5 b	0.34 ± 0.01 ab	7.6 ± 0.1 c
**incl. leaching**				
CTRL-0	0.1 ± 0.1 b	0.6 ± 0.1 d	0.31 ± 0.03 b	7.7 ± 0.1 bc
NPK	1.2 ± 1.3 b	18.8 ± 5.2 c	0.37 ± 0.03 a	8.0 ± 0.1 a
gBBF	1.0 ± 1.6 b	17.9 ± 3.6 c	0.35 ± 0.03 ab	7.8 ± 0.1 ab
B+NPK	4.9 ± 1.8 a	20.0 ± 2.6 c	0.38 ± 0.04 a	7.7 ± 0.1 bc

Table S10 *Statistical results of two-way analyzes of variance for nutrient uptake in aboveground cabbeg biomass. Factors: 'Leaching' (no leaching /incl. leaching), 'Fertilizer type' (NPK/gBBF/B+NPK) and the interaction of both individual factors. Additionally, the block effect is presented 'Block'.*

**Factor**	**Nitrogen uptake**		**Phosphorus uptake**		**Potassium uptake**	
	**F**	**p**	**F**	**p**	**F**	**p**
**Leaching**	0.97	0.3577	11.10	**0.0126**	0.13	0.7263
**Fertilizer Type**	1.97	0.1757	5.24	**0.0214**	0.10	0.9066
**Leaching x Fertilizer Type**	1.11	0.3568	0.22	0.8035	2.17	0.1408
**Block**	1.48	0.2532	1.08	0.4265	0.98	0.4799
**Factor**	**Magnesium uptake**		**Sulfur uptake**		**Calcium uptake**	
	**F**	**p**	**F**	**p**	**F**	**p**
**Leaching**	0.96	0.3380	3.88	**0.0629**	0.05	0.8183
**Fertilizer Type**	7.83	**0.0031**	7.30	**0.0042**	4.10	**0.0322**
**Leaching x Fertilizer Type**	0.82	0.4542	0.52	0.6022	0.67	0.5208
**Block**	0.6841	0.6841	0.6901	0.6796	0.81	0.5963
**Factor**	**Manganese uptake**		**Copper uptake**		**Zinc uptake**	
	**F**	**p**	**F**	**p**	**F**	**p**
**Leaching**	0.90	0.3545	21.61	**0.0002**	1.38	0.2542
**Fertilizer Type**	14.38	**0.0001**	2.86	0.0833	4.03	**0.0346**
**Leaching x Fertilizer Type**	0.47	0.6288	0.35	0.7061	0.05	0.9533
**Block**	0.63	0.7197	0.78	0.6124	0.93	0.5118

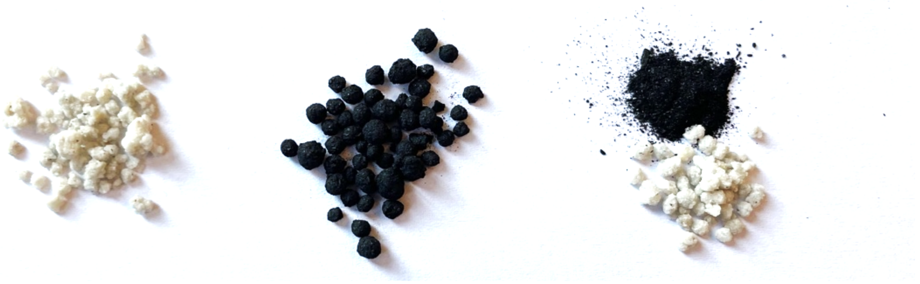

Figure S1 *Fertilizers used in the study: pure granulated mineral fertilizer (NPK,left), granulated biochar-based NPK fertilizer (gBBF, middle), co-application of non-granulated biochar and the granulated NPK fertilizer (B+NPK, right).*

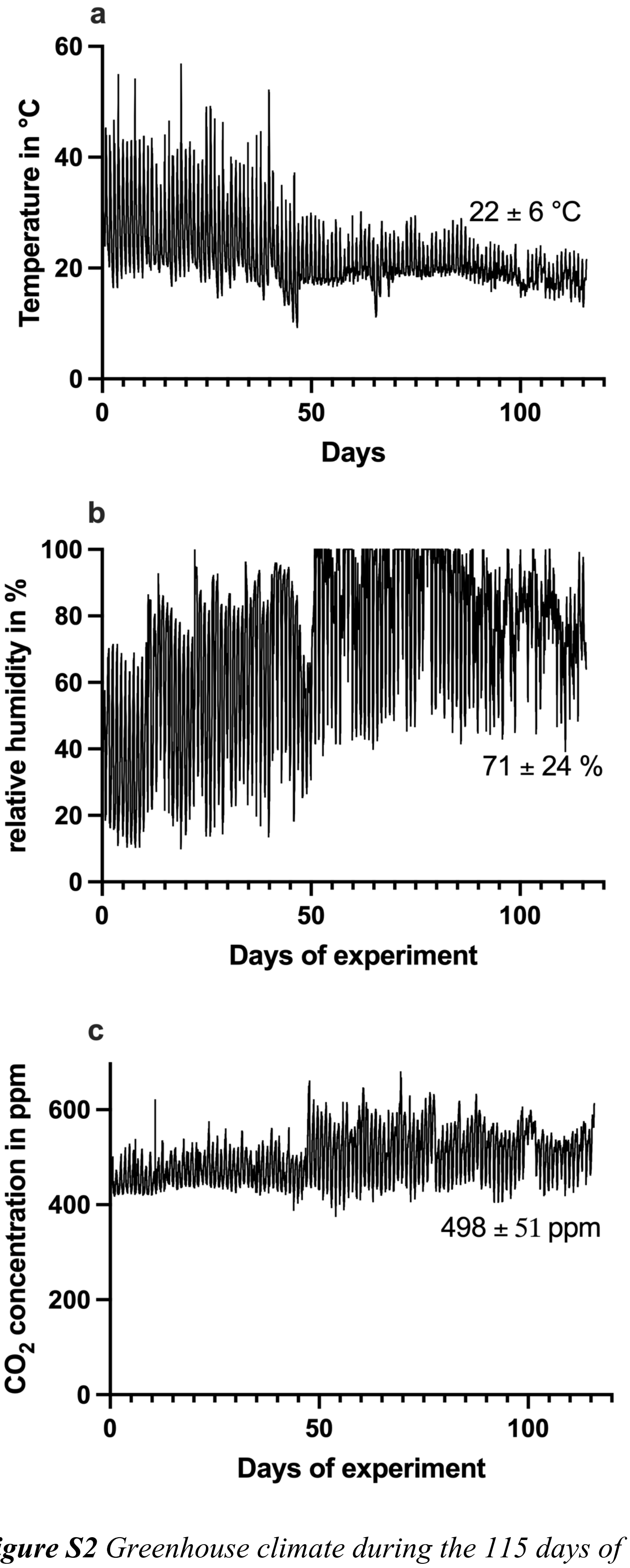

Figure S2 *Greenhouse climate during the 115 days of the pot trial: Temperature (a), relative humidity (b) and ambient* CO_2 *concentration logged in 10-minute intervals (SD800, Extech Instruments, Nashua, USA).*

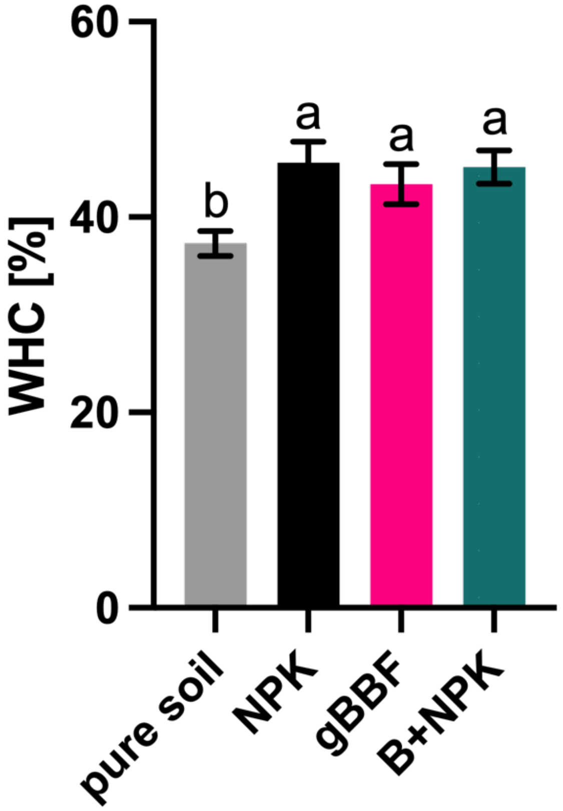

Figure S3 *Water holding capacities (WHC) of the different soil mixtures, expressed as ratio of the dry mixture of soil and fertilizer in %. Data are presented as means ± standard deviation. Different letters above error bars indicate significant differences (one-way ANOVA, $p<0.05$, Tukey's post hoc test).*

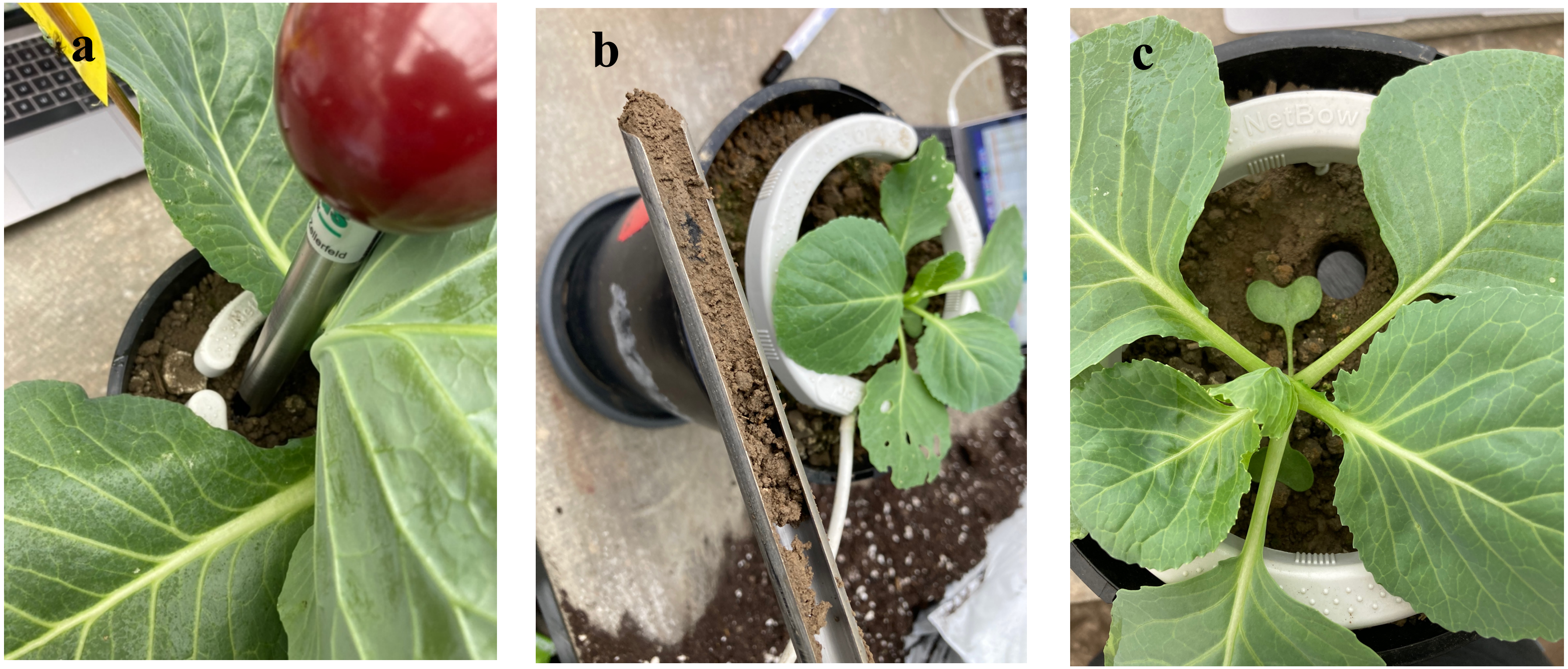

Figure S4 *Soil sampling in redundantly prepared pots for quantification of extractable nitrogen and urease activity in the soil from pots fertilized with NPK, gBBF or B+NPK: soil core sampler inserted in a pot (a), soil core sampled from the pot (b), plastic cylinder inserted into the sampling hole (c).*

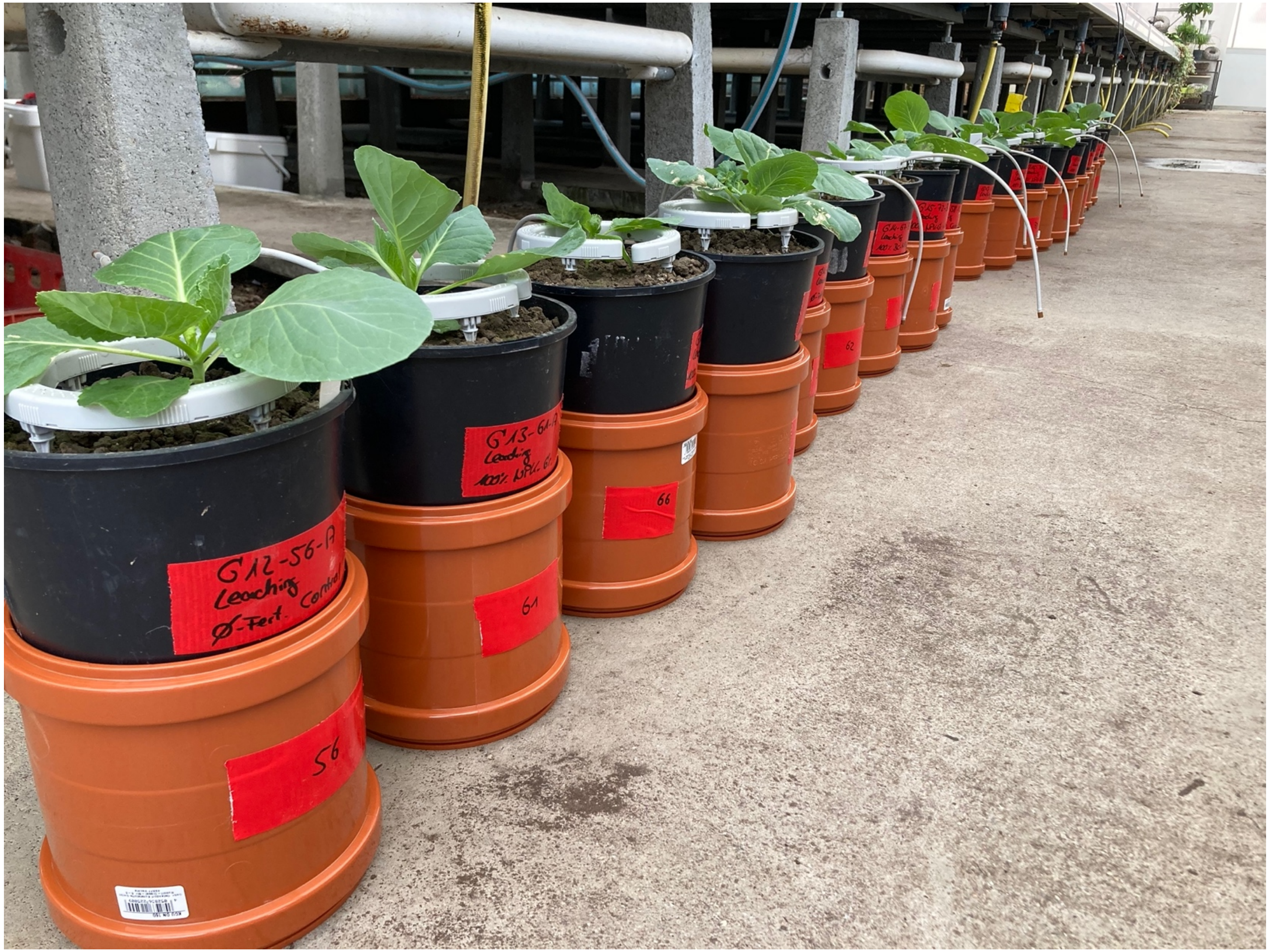

Figure S5 *Pots fixed on bottom-sealed pipe sockets before conduction of precipitation events for nutrient leaching from each individual pot.*

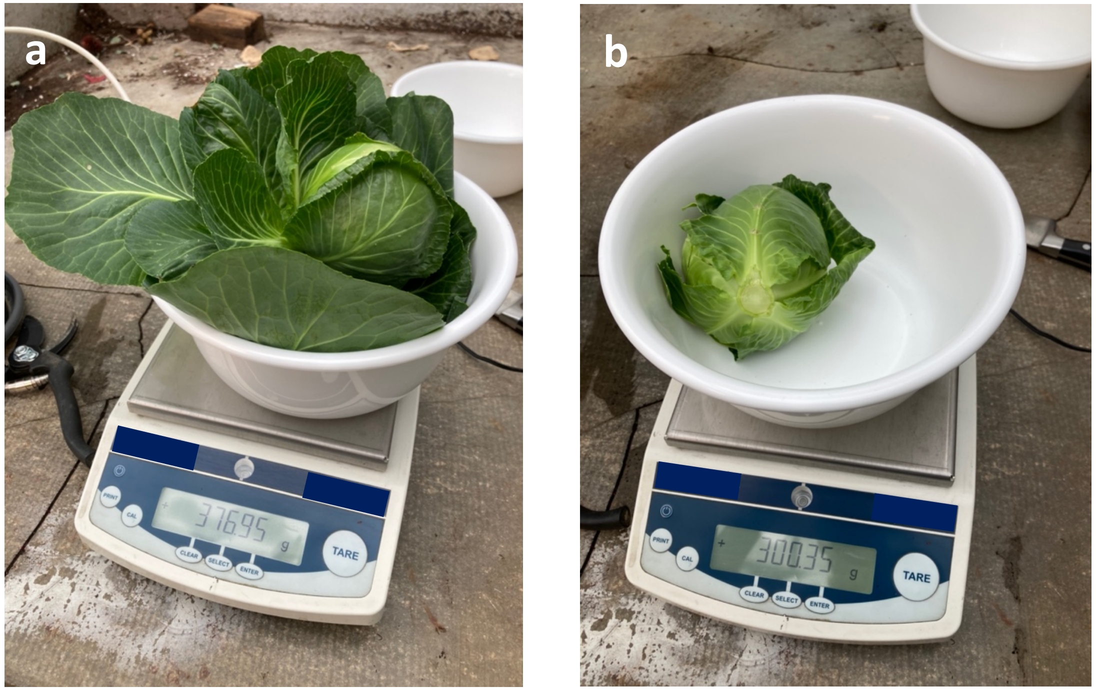

Figure S6 *Aboveground biomass harvested by cutting the cabbage plants below the lowest leaf base (a) and cabbage head separated from marketable, unfolded cabbage leaves (b).*

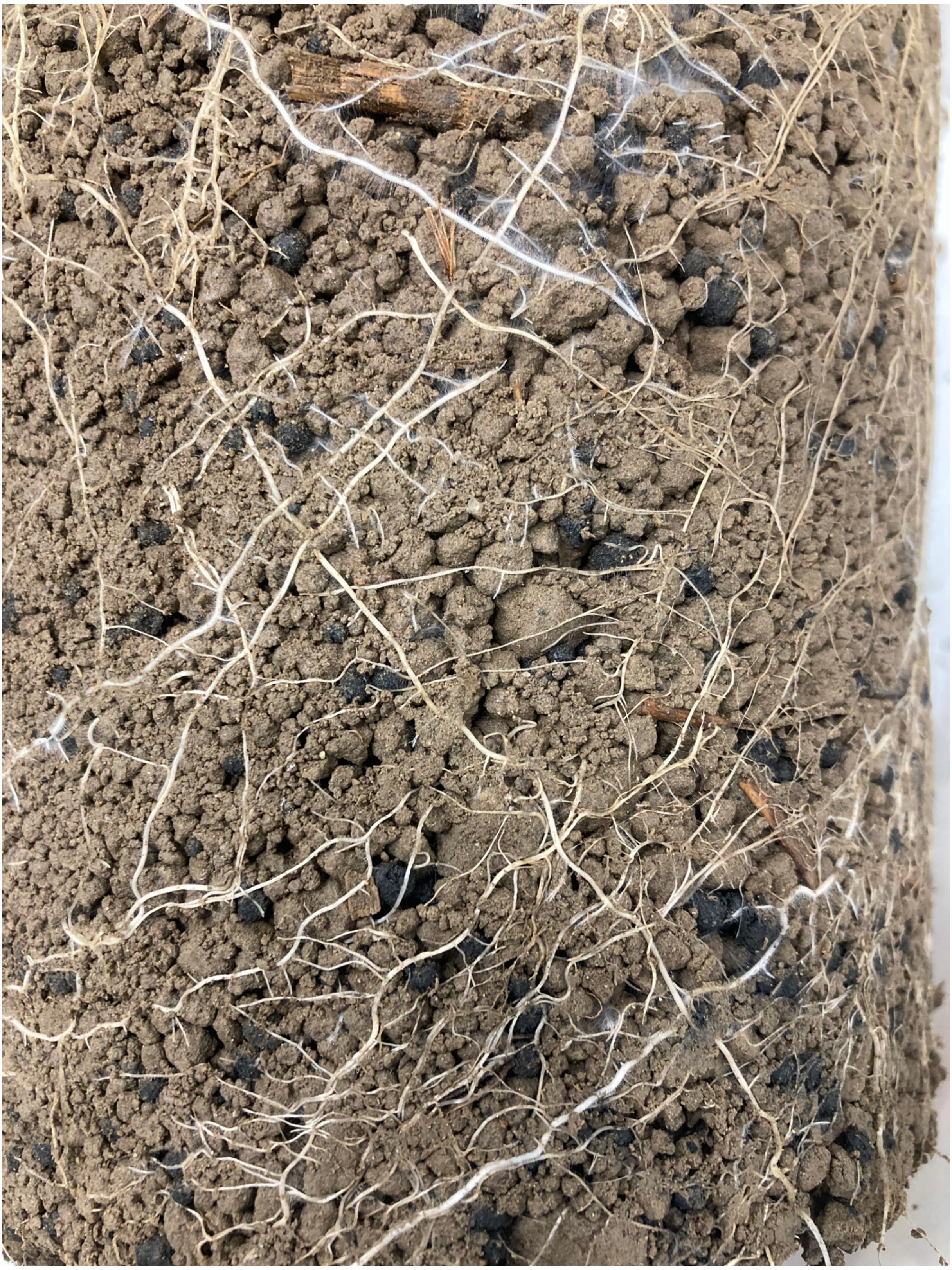

Figure S7 *Intact granules of biochar-based fertilizer (gBBF) after removing the pot from the soil profile before taking (1) removing the rootstock from soil, (2) taking a representative soil sample and (3) taking samples of intact gBBF for later analysis.*

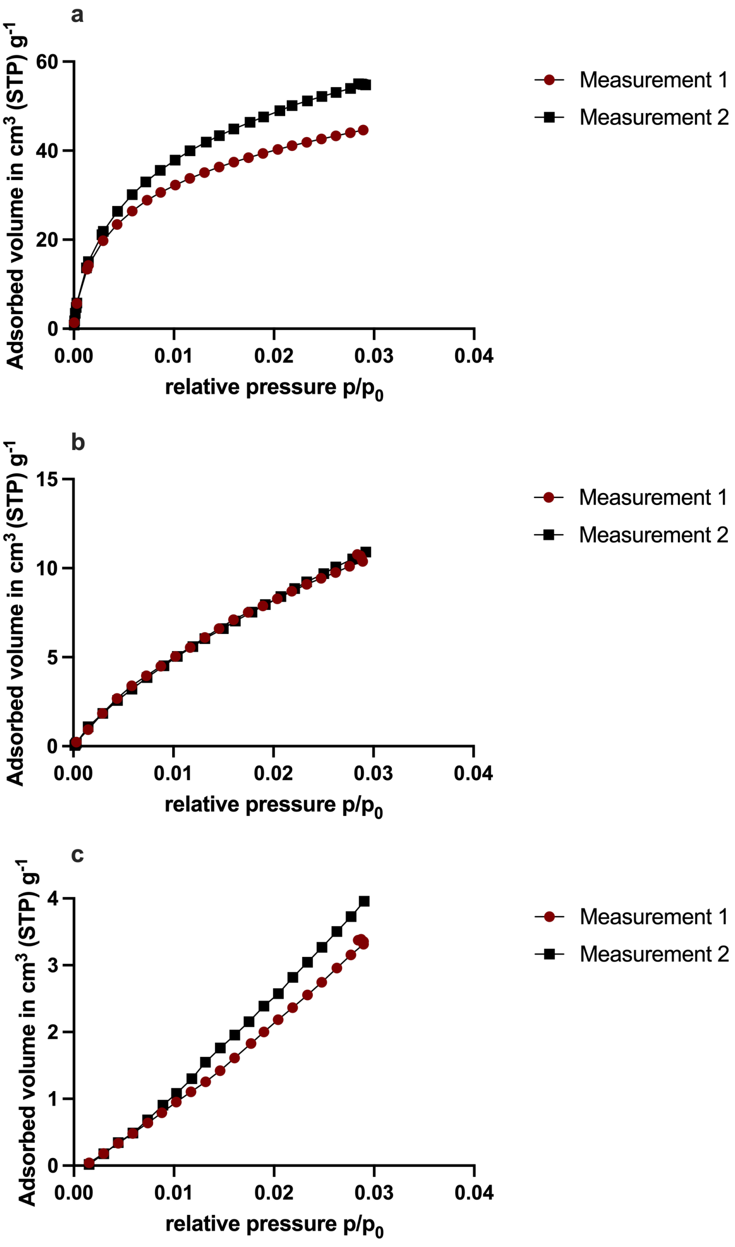

Figure S8 *CO_2 adsorption isotherms of the collard-milled biochar (<1 mm) (a) the granulated biochar-based fertilizer (b) and the granulated NPK fertilizer (c, two replicated measurements). Measurements were performed at 273.15K. Samples were outgassed at 303.15 K under vacuum for 12h before measurements. The adsorbed volume of CO_2 was transformed to standard temperature conditions.*

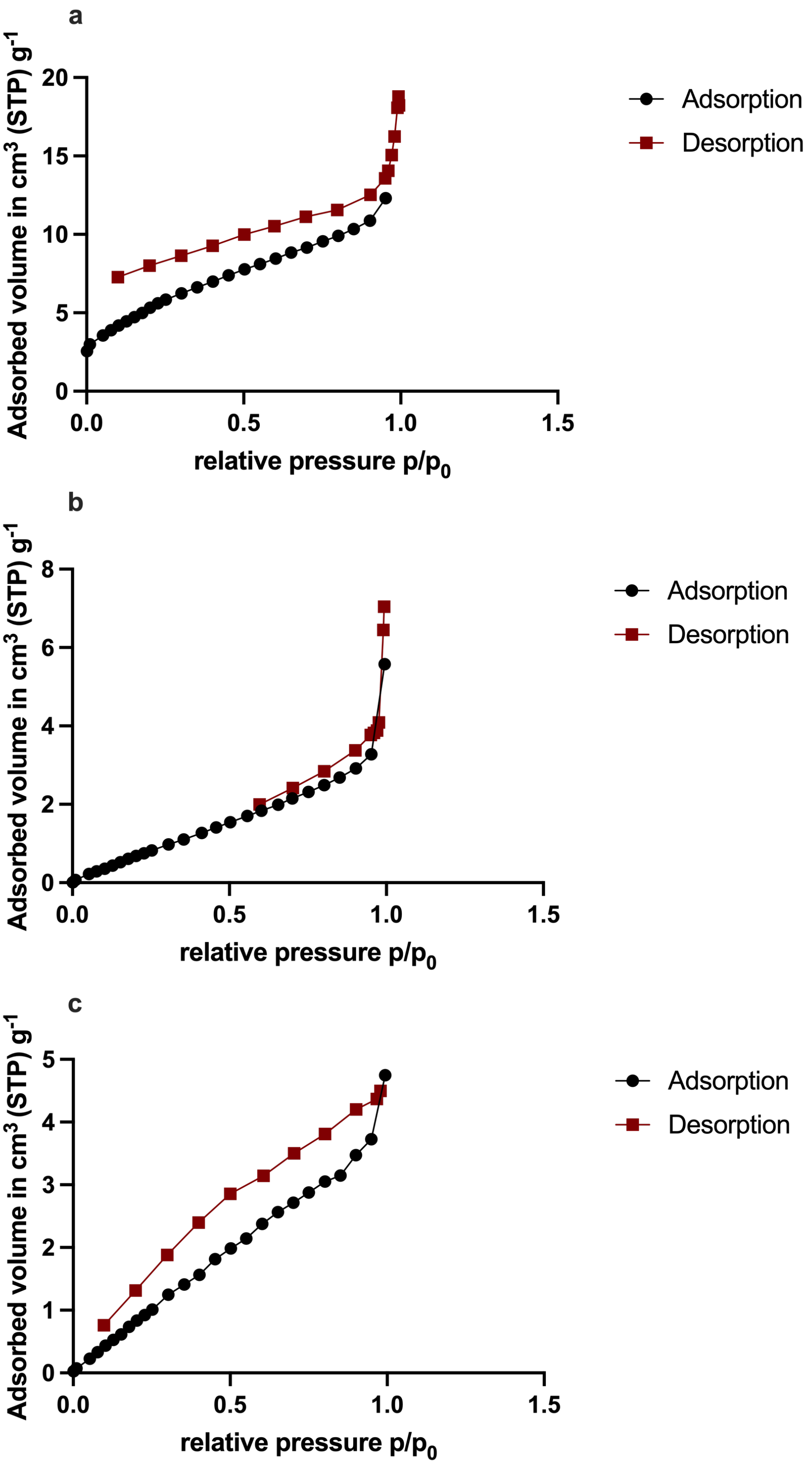

Figure S9 *Nitrogen (N_2) adsorption and desorption isotherms of the collard-milled biochar (< 1mm, a) the biochar-based fertilizer (b) and the mineral NPK fertilizer granule (c). Measurements were performed at 77 K after outgassing the samples for 12 hours at 303.15 K. The adsorbed volume of N_2 was transformed to standard temperature conditions.*

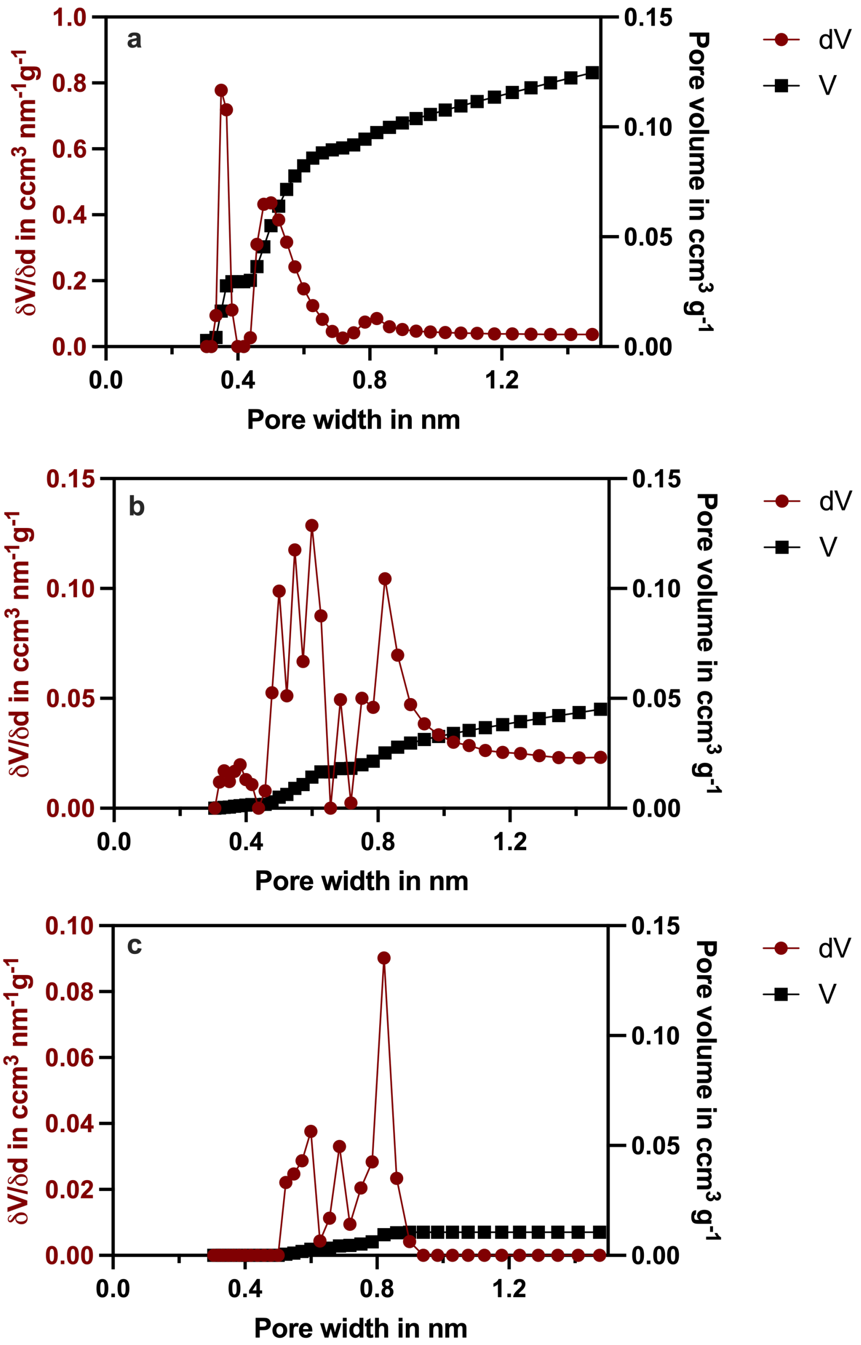

Figure S10 *Pore size distribution of collard-milled biochar (<1 mm, a), the granulated biochar-based fertilizer (gBBF, b) and the granulated mineral fertilizer (NPK, c): First derivate of the pore volume by pore width (δV/δd, red) and cumulative pore volume (black). Data is based on* CO_2 *adsorption experiments performed at 273.15 K after outgassing of the samples at 30°C for 12 h.*

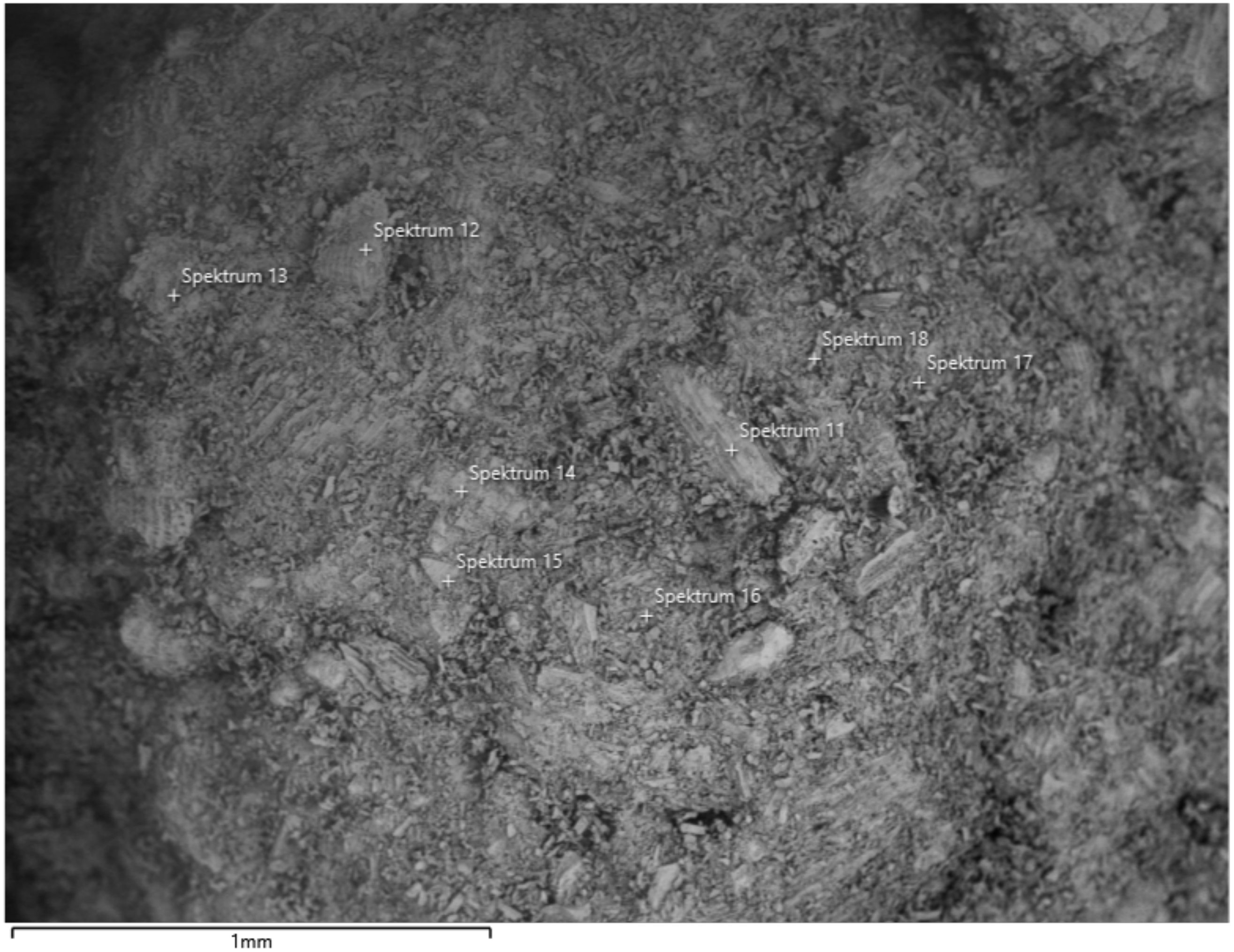

Figure S11 *Scanning electron microscope image of the surface of a granulated biochar-based NPK fertilizer (gBBF). The full length of the scale bar represents a length of 1 mm. 'Spektrum' indicates spots manually picked for elemental analysis by electron dispersive x-ray spectroscopy.*

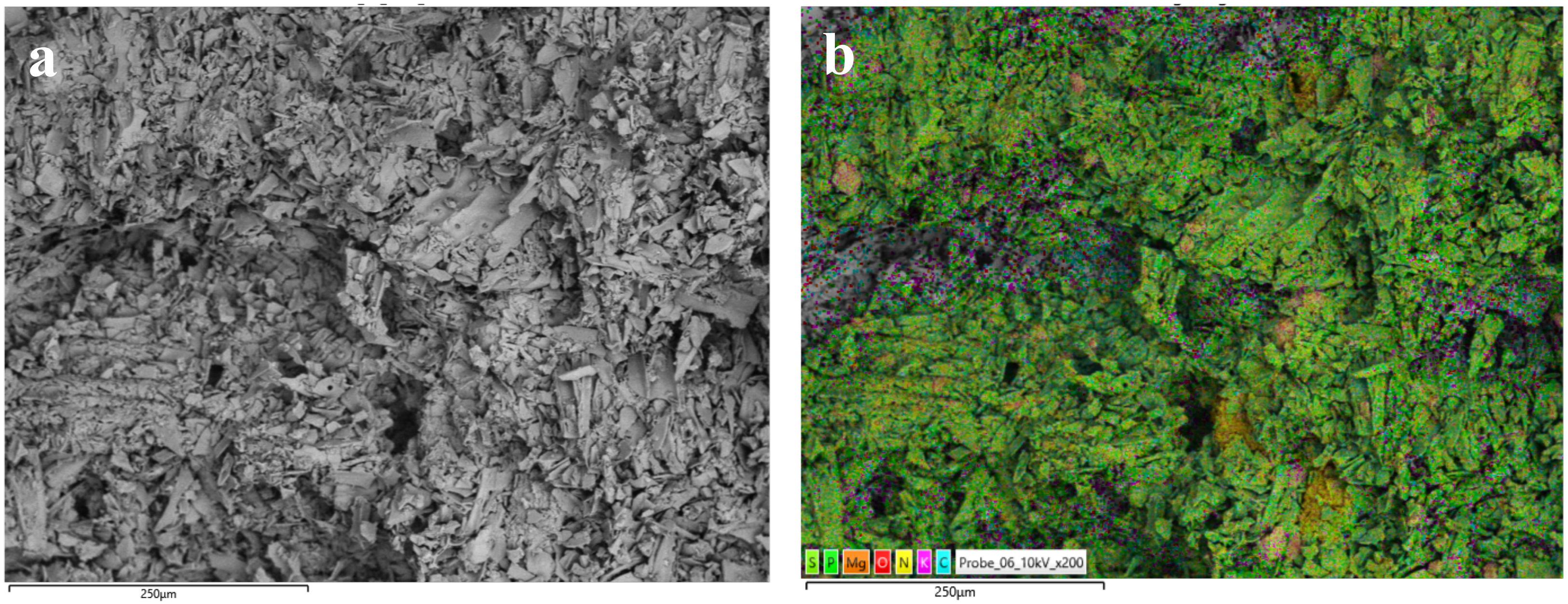

Figure S12 *Scanning electron microscope image (a) and (b) corresponding energy dispersive spectroscopy (EDX) mapping overlay of an interface of a granulated biochar-based NPK fertilizer (gBBF). The granules were sliced with a scalpel before measurements. The full length of the scale bar represents a length of 250 µm. The EDX image measurement was acquired with 10 kV accelerating voltage. Mapped elements are carbon (C), oxygen (O), sulfur (S), phosphorus (P), potassium (K), magnesium (Mg) and nitrogen (N). The mapping of the individual elements can be found in Figure S13.*

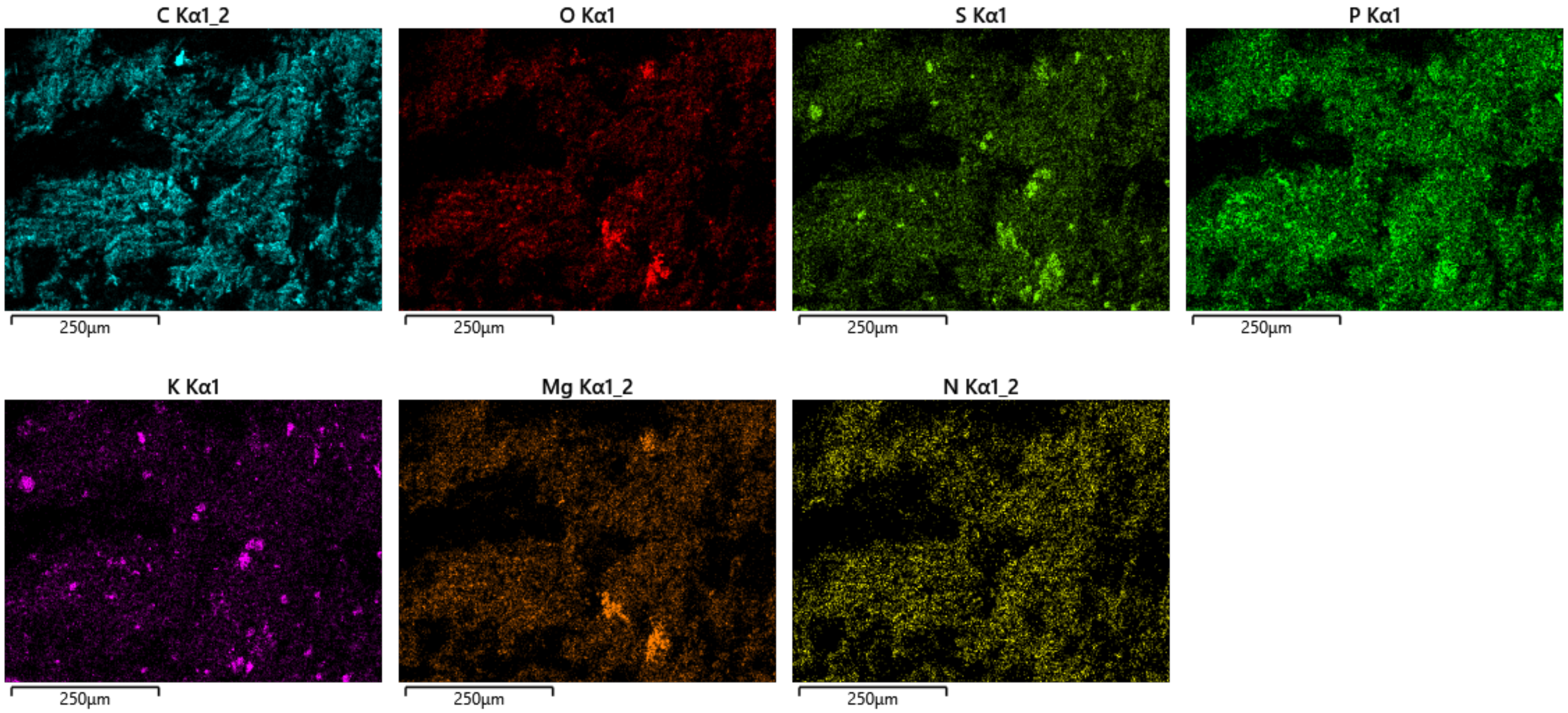

Figure S13 *Energy dispersive spectroscopy (EDX) mapping on an interface of a granulated biochar-based NPK fertilizer (gBBF). The original scanning electron microscope image and the overlay of the EDX mapping can be found in Figure S12. Mapped elements are carbon (C), oxygen (O), sulfur (S), phosphorus (P), potassium (K), magnesium (Mg) and nitrogen (N). Images were obtained with an accelerating voltage of 10 kV.*

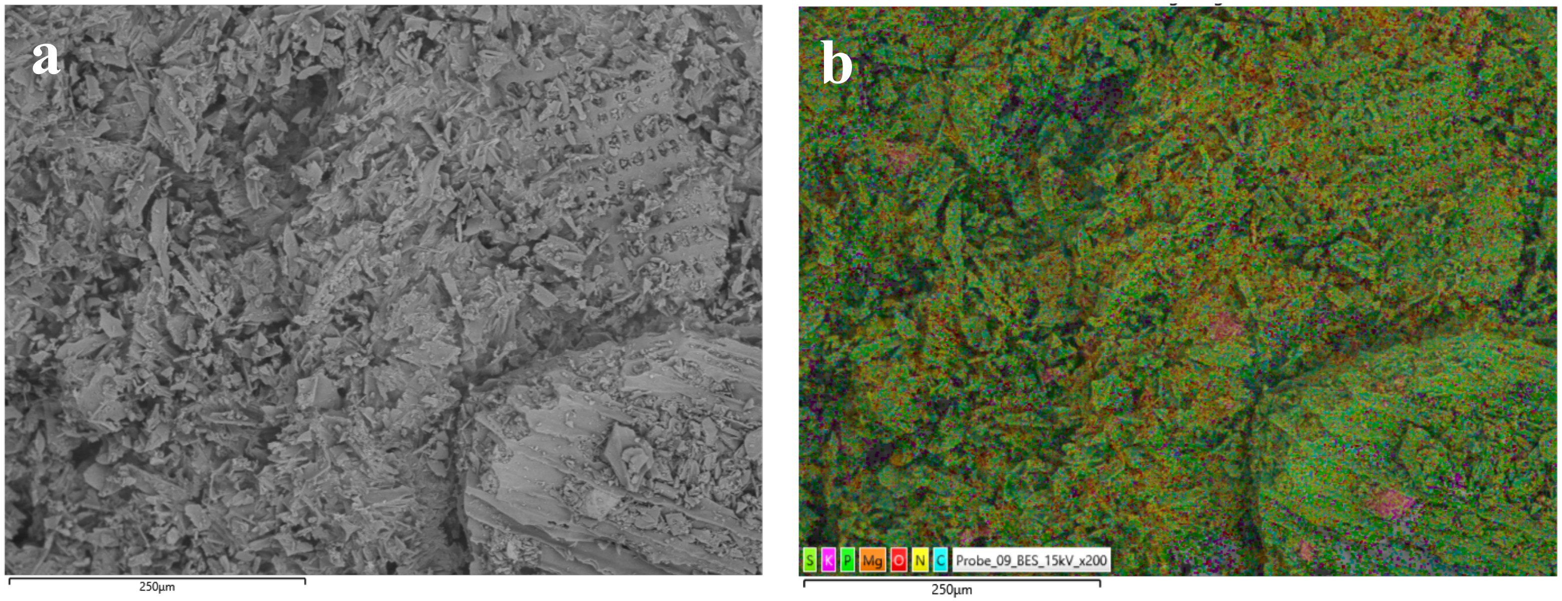

Figure S14 *Scanning electron microscope image (a) and corresponding energy dispersive spectroscopy (EDX) mapping overlay of an interface of a granulated biochar-based NPK fertilizer (gBBF). The granules were sliced with a scalpel before measurements. The full length of the scale bar represents a length of 250 µm. The EDX image measurement was acquired with 15 kV accelerating voltage. Mapped elements are carbon (C), oxygen (O), sulfur (S), phosphorus (P), potassium (K), magnesium (Mg) and nitrogen (N). The mapping of the individual elements can be found in Figure S15.*

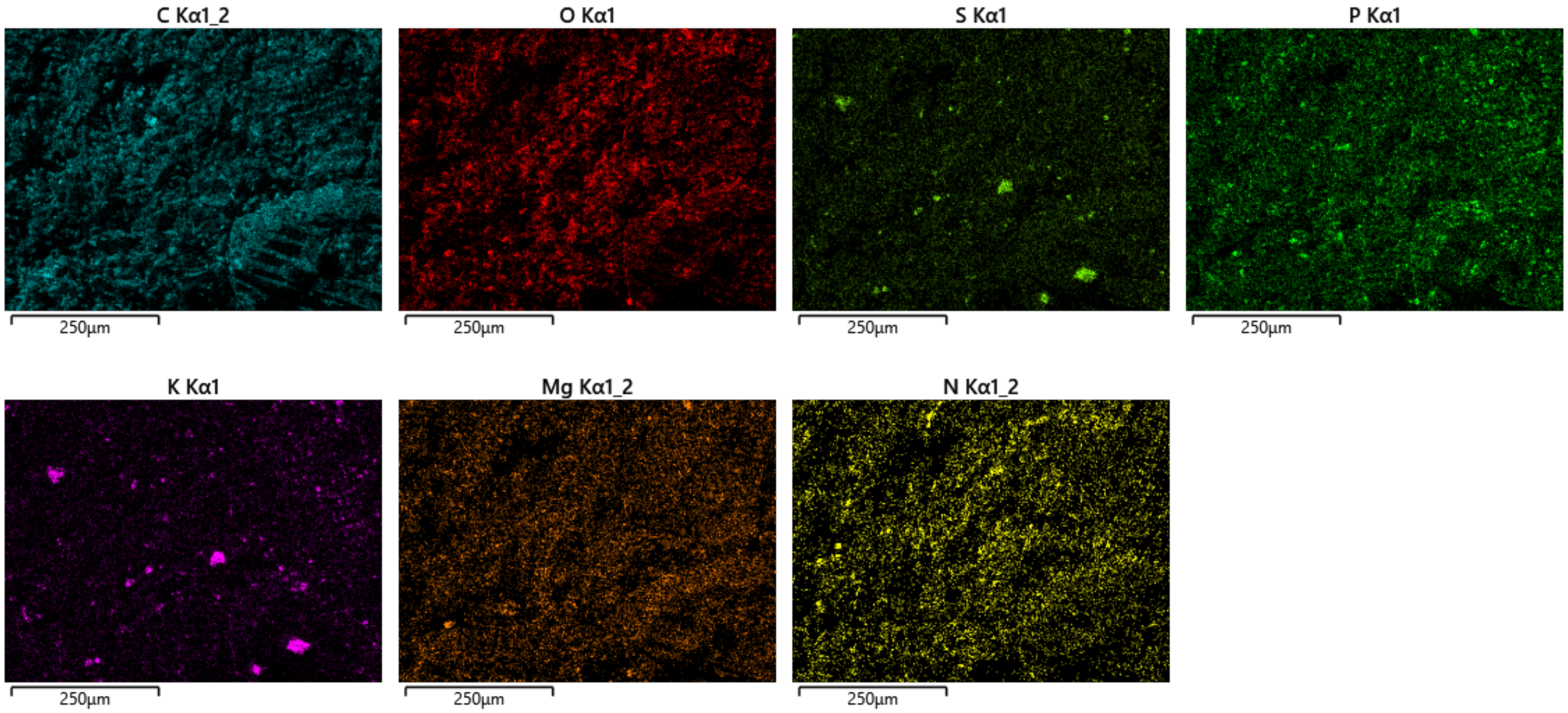

Figure S15 *Energy dispersive spectroscopy (EDX) mapping on an interface of a granulated biochar-based NPK fertilizer (gBBF). The original scanning electron microscope image and the overlay of the EDX mapping can be found in Figure S14. Mapped elements are carbon (C), oxygen (O), sulfur (S), phosphorus (P), potassium (K), magnesium (Mg) and nitrogen (N). Images were obtained with an accelerating voltage of 15 kV.*

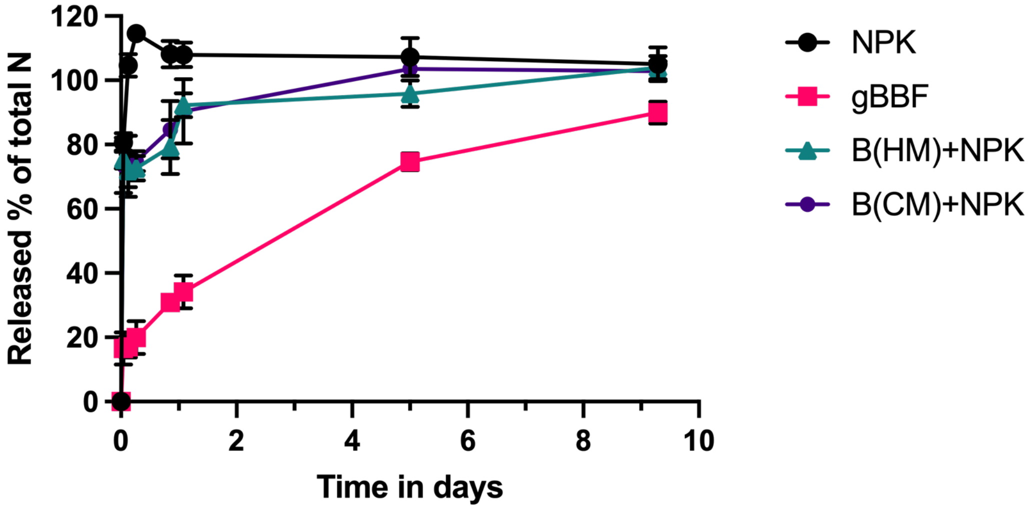

Figure S16 *Release of nitrogen (N) from the fertilizer granules and the mixture of the NPK fertilizer with milled biochar in non-shaken distilled water during 9 days. Error bars show the standard deviation (n= 3). Released N is presented as percentage of total N in the respective fertilizer mixtures. NPK: pure NPK fertilizer granule; gBBF: granulated biochar-based fertilizer; BC(HM)+NPK: loose mixture of hammer milled biochar (<12 mm) and pure NPK fertilizer; BC(CM)+NPK: loose mixture of collard milled biochar (< 1mm) and pure NPK fertilizer. For the loose mixture of BC and NPK, the materials were separately weighted into the incubation bottle before addition of the distilled water.*

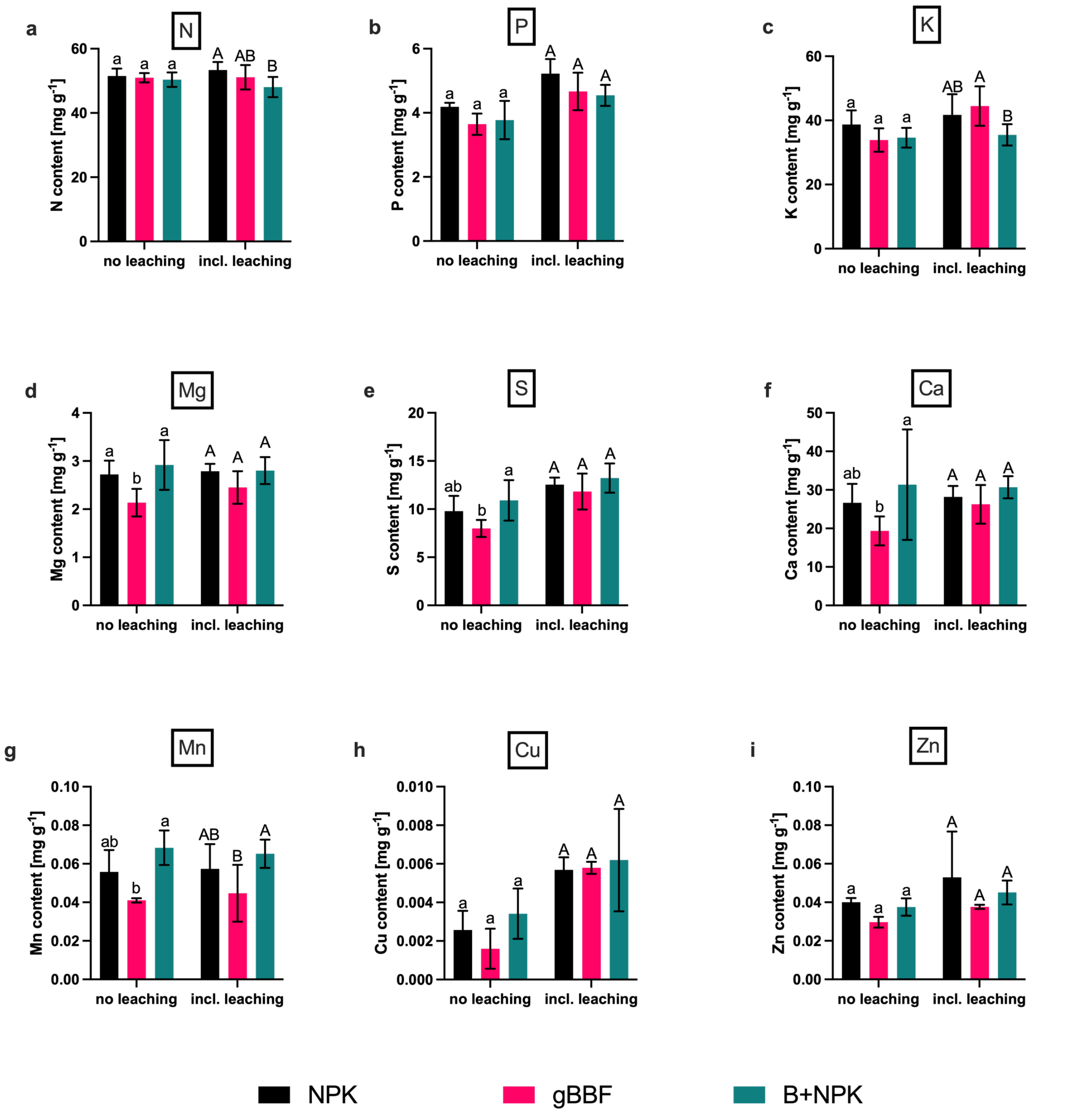

Figure S17 *Nutrient contents in aboveground tissue of white cabbage plants: nitrogen (N) potassium (K), phosphorus (P), magnesium (Mg). sulfur (S), calcium (Ca), manganese (Mn), copper (Cu) and zinc (Zn). The plants were grown either with a granulated NPK fertilizer (NPK), a granulated biochar-based NPK fertilizer (gBBF) or with the amendment of non-granulated biochar combined with pure NPK fertilizer (B+NPK). Data is presented as mean ± standard deviation (n=5). Different letters above error bars indicate a statistically significant difference each within the 'no leaching' or the 'including leaching' treatments (lowercase and uppercase letters, respectively, two-way ANOVA and Tukey's post-hoc test, p<0.05).*

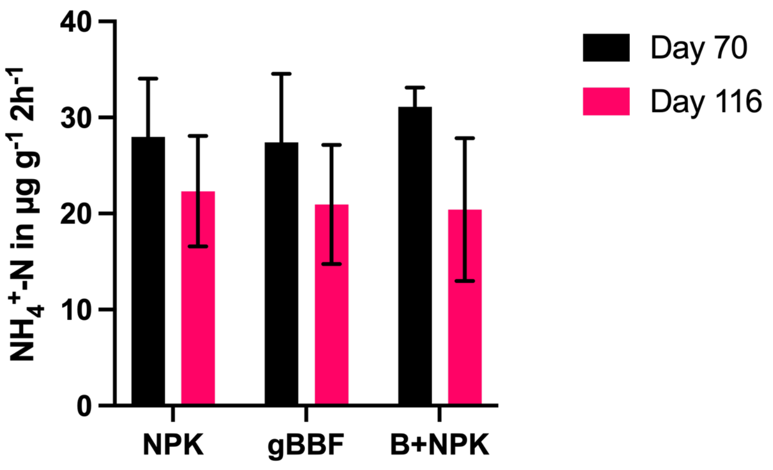

Figure S18 *Soil urease activity in soil samples taken in redundantly prepared pots after 70 days and after harvest. For the measurement after 70 days, five redundantly prepared pots were sampled with a soil core sampler. The samples at day 116 were obtained during harvest after separation of the cabbage rootstocks from the soil. Data is presented as mean ± standard deviation (n=5). NPK: granulated NPK fertilizer. gBBF: granulated biochar-based NPK fertilizer. B+NPK: co-application f biochar and granulated NPK fertilizer.*

a

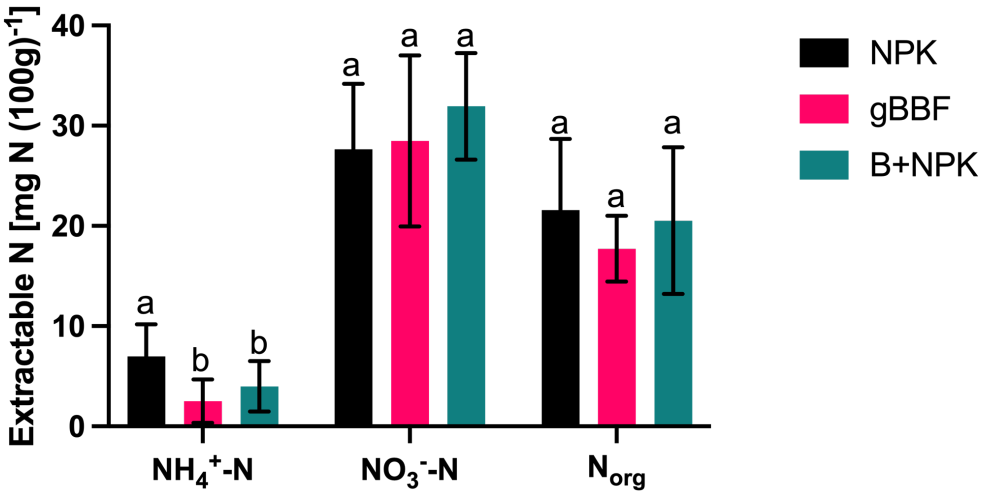

b

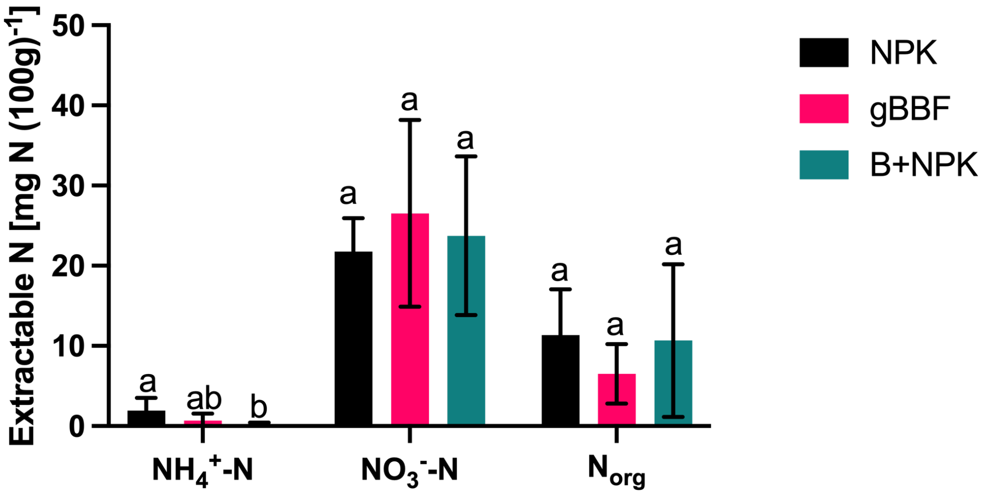

Figure S19 *Extractable nitrogen (N) species in the soil samples taken after 36 days (panel a) and 70 days (panel b) after sowing. The pots were redundantly prepared and on the specified days, one soil sample per pot was taken with a soil core sampler through the whole pot depth. These pots did not receive leaching events. Extractable total N,* NO_3^-*-N and* NH_4^+*-N was measured in filtered 0.0125M* $CaCl_2$ *extracts (1+4; m+V). The difference between total N and the two measured mineral N species was defined as organic N (*N_{org}*). Data are presented as mean ± standard deviation (n=5). The data were tested for significant differences by one-way analysis of variance for each N fraction individually (*$p<0.05$*, Tukey's post-hoc test, indicated by different letters above error bars).*

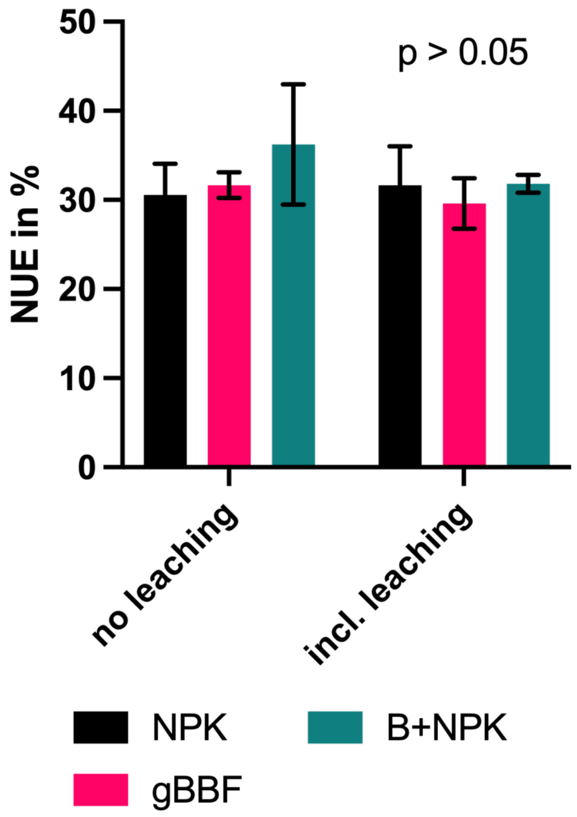

Figure S20 *Nitrogen use efficiency (NUE) of all fertilized plants based on the N uptake in the aboveground biomass with or without leaching events. Data is presented as mean ± standard deviation (n=5). NPK: pure NPK fertilizer. gBBF: biochar-based NPK fertilizer granule. B+NPK: Milled biochar applied to the soil in combination with pure NPK fertilizer. The non-significant p value was obtained from two-way analysis of variance ($p<0.05$).*

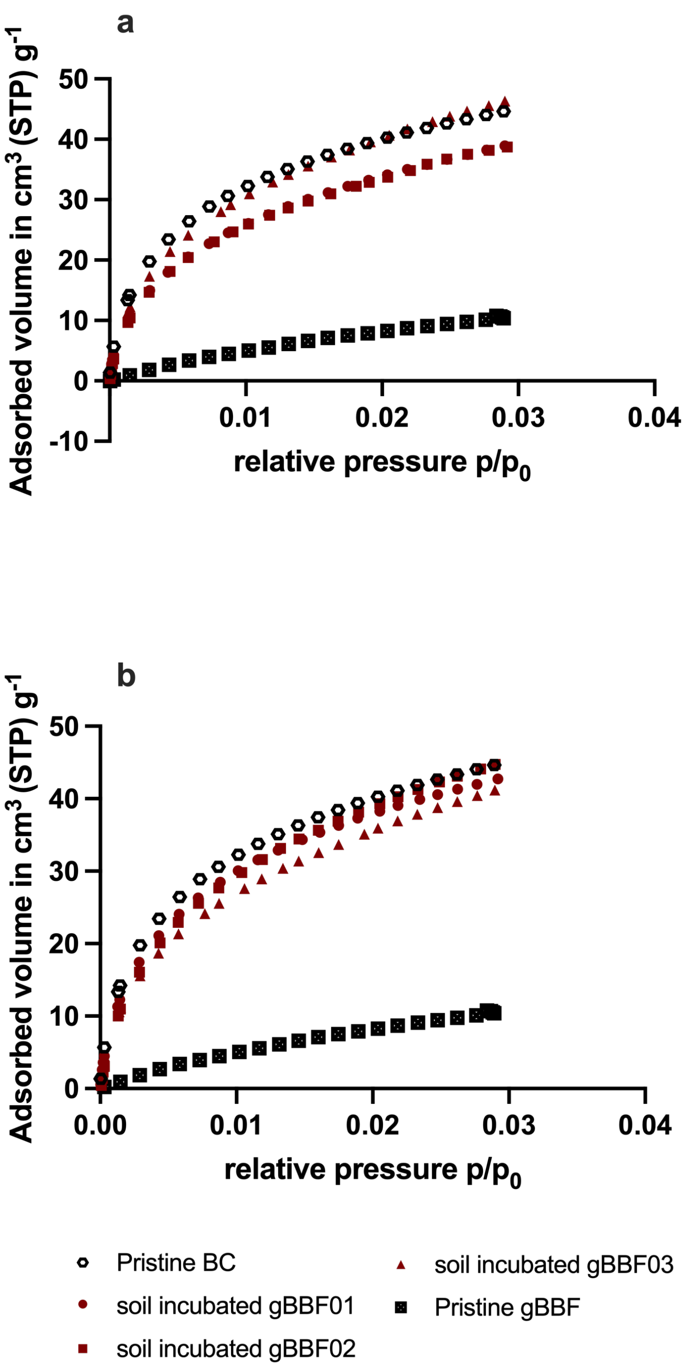

Figure S21 *CO_2 adsorption isotherms recorded for the pristine biochar (pristine BC), the pristine granulated biochar-based fertilizer (pristine gBBF) and soil aged gBBF granules (three replicates labelled as 'soil incubated gBBF'), either sampled from pots without (a) or with (b) leaching events during cultivation of white cabbage. Measurements were performed at 273.15K. Samples were outgassed at 303.15 K under vacuum for 12h before measurements. The adsorbed volume of CO_2 was transformed to standard temperature conditions.*

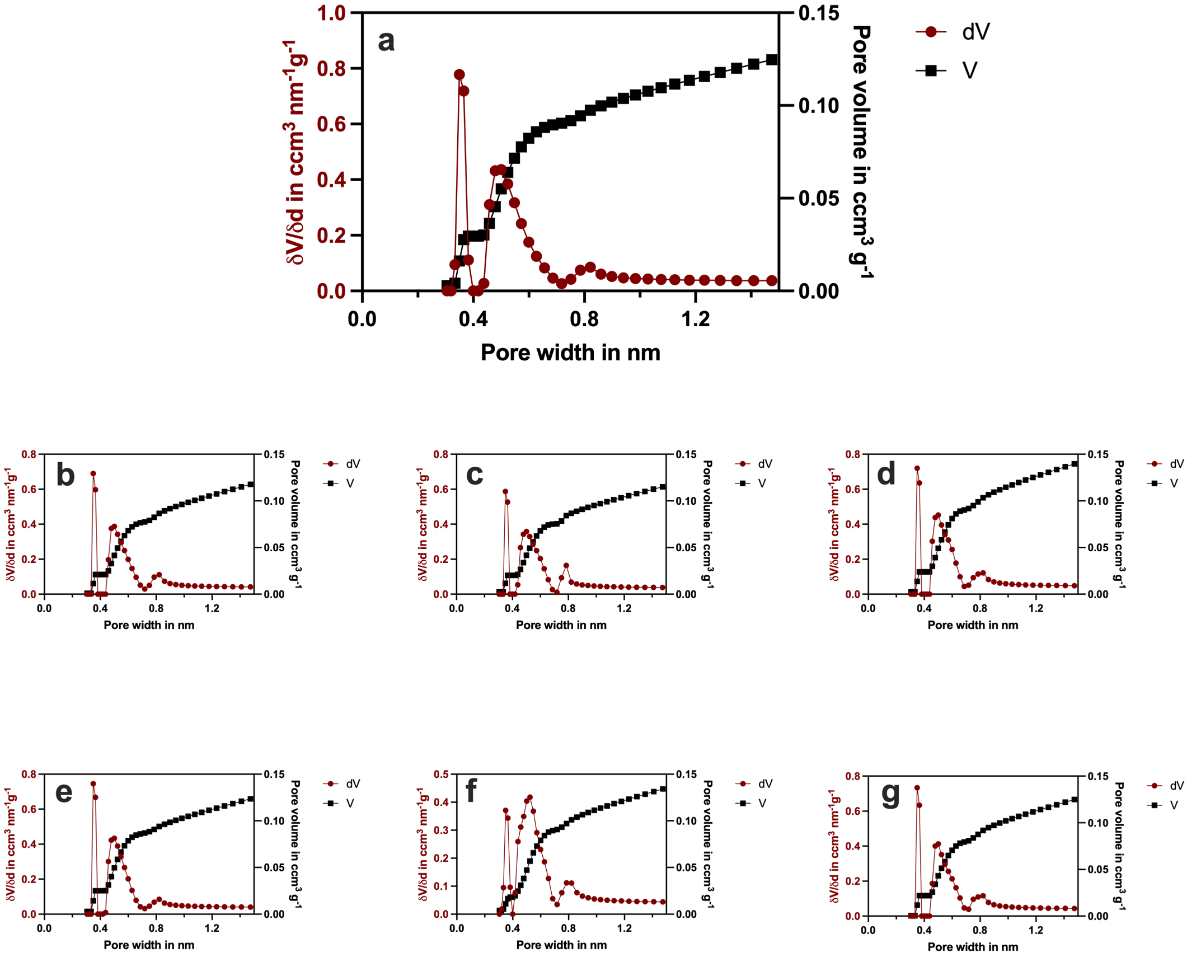

Figure 22 *Pore size distribution of pristine collard-milled biochar (BC, a), soil-aged gBBF granules sampled after harvest of cabbage plants in pots without leaching events (b-d) and soil-aged gBBF granules sampled in pots including leaching events (e-g). The first derivate of the pore volume by pore width (δV/δd, red) and cumulative pore volume (black) is displayed. Data is based on CO_2 adsorption experiments performed at 273.15 K after outgassing of the samples at 30°C for 12 h.*

References

1. Tahery, S. *et al.* A comparison between the characteristics of a biochar-NPK granule and a commercial NPK granule for application in the soil. *Sci. Total Environ.* **832**, 155021 (2022).

2. Kandeler, E. & Gerber, H. Short-term assay of soil urease activity using colorimetric determination of ammonium. *Biol. Fertil. Soils* **6**, (1988).

3. Rhine, E. D., Mulvaney, R. L., Pratt, E. J. & Sims, G. K. Improving the Berthelot Reaction for Determining Ammonium in Soil Extracts and Water. *Soil Sci. Soc. Am. J.* **62**, 473 (1998).

4. Muehe, E. M., Wang, T., Kerl, C. F., Planer-Friedrich, B. & Fendorf, S. Rice production threatened by coupled stresses of climate and soil arsenic. *Nat. Commun.* **10**, 4985 (2019).

5. Hagemann, N. *et al.* Does soil aging affect the N_2O mitigation potential of biochar? A combined microcosm and field study. *GCB Bioenergy* **9**, 953–964 (2017).
